# Reliable AI Platform for Monitoring BCI Caused Brain Injury and Providing Real‐Time Protection

**DOI:** 10.1002/advs.202506747

**Published:** 2025-12-24

**Authors:** Chufan He, Yanjun Ding, Timon Rabczuk, Chensen Ding

**Affiliations:** ^1^ School of Mechanics and Engineering Science Peking University Beijing 100871 China; ^2^ Department of Forensic Medicine, Xiangya School of Basic Medical Sciences Central South University Changsha 410013 China; ^3^ Institute of Structural Mechanics Bauhaus University Weimar 99423 Weimar Germany

**Keywords:** AI platform for Real‐time monitoring and protection, brain computer interface, brain injury, digital brain twin, Gaussian process regression

## Abstract

Invasive brain‐computer interface (BCI) holds great promise for restoring motor, sensory, and cognitive functions in patients with disabilities, yet chronic implantation induces neuroinflammation and degeneration at the electrode–tissue interface, impairing neural connectivity and device long‐term stability. Current brain injury assessment approaches cannot simultaneously meet the requirements of efficiency and interpretability in healthcare with high‐risk diagnoses and treatment. Meanwhile, limited and expensive biomechanics data pose significant challenges in AI training. Herein, feature‐based Gaussian process emulators are proposed to enable interpretable data‐driven modeling with limited biomechanics data under noise. Furthermore, a reliable AI platform, BrainGuard is developed, for efficiently providing a reliable and quantitative patient‐specific basis and real‐time monitoring of BCI caused brain injury. These results demonstrate exceptional performance of BrainGuard in rapidly and accurately predicting and monitoring the full‐field von Mises strain revealing the brain injury even under challenging noise conditions. By constructing interpretable digital brain twins to offer reliable digital healthcare solutions, the platform enhances real‐time patient protection and improves the security and durability of long‐term BCI‐based measurement and treatment strategies.

## Graphical abstract

BrainGuard enables real‐time and interpretable assessment of brain injury caused by brain computer interface (BCI). Using feature‐based Gaussian process (GP) emulators trained on limited biomechanical data, it efficiently predicts full‐field strain and constructs patient‐specific digital brain twins to support clinical diagnosis and long‐term BCI safety.

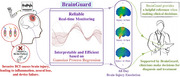

## 1 Introduction

The brain computer interface (BCI)^[^ ^1^, ^2^, ^3^, ^4^, ^5^ ^]^ captures electrophysiological signals transmitted between the brain neurons, and creates a direct communication pathway between brain electrical activity and external devices such as computers or robotic limbs, repairing the sensory‐motor functions, treating neurological diseases, controlling unmanned aerial vehicles, virtual and augmented reality, etc. Invasive BCI^[^ ^6^, ^7^, ^8^ ^]^ offers high‐definition and high‐resolution signal collection, facilitating precise machine control and disease treatment at the single‐neuron level. By implanting these devices into the cerebral cortex, they significantly reduce signal attenuation caused by the skull and scalp, thereby enhancing the accuracy and effectiveness of the interfaces. For example, Neurolink's first patient with quadriplegia is able to control the computer directly to play games for 8 h straight after recovering from BCI implantation surgery,^[^ ^9^, ^10^ ^]^ reshaping the treatment of disable patients. Several long‐term clinical studies, including BrainGate trials^[^ ^11^ ^]^ and a 5‐year follow‐up of a fully implanted ECoG system in a patient with spinal cord injury,^[^ ^12^ ^]^ have shown that signal quality metrics (such as SNR, electrode impedance, decoder accuracy) remain relatively stable over many months/years under real‐world conditions. These studies suggest that invasive BCIs are not only evolving rapidly in capability but are also increasingly validated by performance over extended durations.^[^ ^13^ ^]^

**Figure 1:**
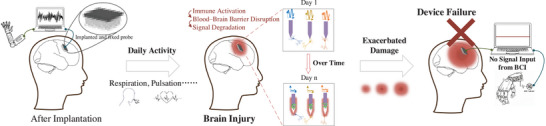
Micromotion‐induced tissue responses and their impact on BCI performance. During normal physiological activities such as respiration and vascular pulsation, micromotion between implanted and fixed probes and brain tissue induces mechanical strain at the electrode–tissue interface. This strain activates microglia and astrocytes, disrupts the blood–brain barrier, and triggers chronic inflammation, leading to neuronal apoptosis and demyelination. Over time (Day n), glial proliferation and sheath formation progressively isolate the recording sites from neurons, degrading signal quality and recording stability. These cumulative biological responses exacerbate local tissue remodeling and ultimately result in signal loss and functional failure of the BCI system.

However, research has found that brain micromotion during respiration, vascular pulsation in daily activity, will activate microglia and astrocytes,^[^ ^14^ ^]^ disrupts the blood–brain barrier,^[^ ^15^, ^16^ ^]^ and triggers neuronal apoptosis and demyelination,^[^ ^17^ ^]^ ultimately leading to chronic neurodegeneration and deterioration of neural recording performance^[^ ^18^, ^19^, ^20^ ^]^ (**Figure** **1**). Particularly, the BCI caused brain injury is primarily driven by high levels of strain fields induced by sustained relative motions between brain tissue and implanted probe fixed on skull.^[^ ^21^, ^22^, ^23^ ^]^ Experimental data from real‐world clinical settings offer essential insights into the quantification of complex physiological processes underlying brain injury and serve as a critical foundation for the validation of computational models. Whereas, stringent ethical regulations, coupled with concerns for patient safety, make the acquisition of such high‐quality data exceedingly difficult in practice.

As a reliable alternative, numerical simulations—particularly those that compute strain fields induced by brain micromotion following BCI implantation^[^ ^24^, ^25^, ^26^, ^27^ ^]^ —have proven effective in quantifying brain injury. These simulations enable the evaluation of BCI‐induced effects and contribute to improving the long‐term stability and safety of BCI.^[^ ^28^, ^29^ ^]^ Nevertheless, high‐fidelity simulations are too computationally expensive to be applied in real‐time uncertainty quantification and digital twins.^[^ ^30^, ^31^, ^32^, ^33^ ^]^ Specifically, when treating different patients, due to patient‐specific, traditional methods require repetitive and time‐consuming finite element calculations, even though, for this series of models, only some parameters are different.^[^ ^34^, ^35^ ^]^

**Figure 2:**
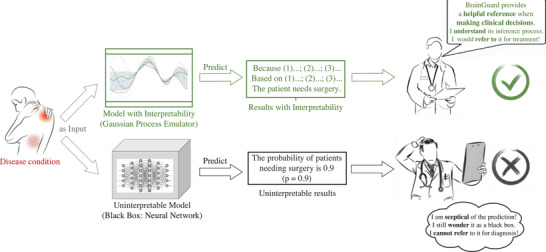
The reliable and unreliable models in healthcare. The proposed framework can predict with interpretability, providing a helpful reference in medical decision‐making. Black box models are difficult to be trust by clinician due to lack of interpretability.

In contrast to high‐fidelity simulations, artificial intelligence (AI) models provide a highly efficient prediction as the most computational effort is invested in the training phase.^[^ ^36^, ^37^, ^38^ ^]^ In healthcare, artificial intelligence methods, which can improve delivery of care at a reduced cost, are gaining widespread application.^[^ ^39^, ^40^, ^41^ ^]^ Artificial neural networks, in particular, possess advantageous characteristics such as self‐learning and adaptability, and the flexibility to perform both linear and nonlinear mapping. These features have facilitated their application in various domains including clinical diagnosis, cancer prediction, speech recognition, and drug development,^[^ ^42^, ^43^, ^44^ ^]^ and have been integrated into decision support models to offer cost‐effective solutions for time and resource management in healthcare systems and providers.^[^ ^43^ ^]^ Nevertheless, neural networks face limitations such as a lack of interpretability, which constrains their application in healthcare, particularly in high‐risk diagnosis and treatment scenarios where reliability and safety are crucial,^[^ ^45^, ^46^ ^]^ making it challenging for clinicians to understand, trust, and make medical decisions (**Figure** **2**). Meanwhile, the lack of biomechanics data, primarily due to the difficulty in obtaining experimental data and the high cost of numerical simulations, poses significant challenges for training neural networks.^[^ ^79^, ^80^ ^]^ Furthermore, neural networks lack a rigorous theoretical framework for inherently managing noise and uncertainty, often necessitating increased model complexity and substantial computational resources. As a result, they face significant challenges in effectively handling data with high noise levels.^[^ ^89^, ^90^ ^]^ These challenges are particularly evident in high‐risk fields such as brain injury prediction and simulation, where data scarcity and varying levels of inherent noise within the data within data directly impacts model performance.^[^ ^80^, ^81^ ^]^

Herein, we first propose feature‐based Gaussian process emulators to enable interpretable data‐driven modeling for efficient brain injury prediction with limited biomechanics data under various noise levels. Building on this foundation, we further develop a novel and reliable AI platform, BrainGuard, designed to build reliable digital brain twins to provide the quantitative, patient‐specific reference and real‐time monitoring of brain injuries caused by BCI due to brain micromotion. BrainGuard employs a feature space construction method that preserves the key features of full‐field von Mises strains, a critical indicator of brain injury, while minimizing information loss through model reduction techniques. From a Bayesian perspective,^[^ ^47^, ^48^ ^]^ BrainGuard utilizes Gaussian Process emulator, which offers improved interpretability compared to other methods and enables to provide more reliable and safer reference for medical decision‐making (Figure 2), to map inputs to patient‐specific strain features, enhancing performance in high‐dimensional predictions across different functional modules with naturally noise estimations and processing. Unlike time‐consuming finite element simulations, BrainGuard enables efficient prediction and real‐time monitoring by leveraging a trained model using a high‐fidelity dataset generated by nonlinear finite element. Based on the established posterior, it can efficiently emulate full‐field von Mises strains, comprising over 300000 points, for new inputs such as patient brain and probe mechanical properties or brain micromotions. Results demonstrate that under various noise levels, BrainGuard rapidly and accurately predicts and monitors brain injuries, with a time consumption of only 0.22 s—less than 1/1600 of numerical simulations—and relative errors in full‐field strains of less than 5 × 10^−3^. This platform builds a trustworthy digital twin for patient's physical brain to offer a supportive digital healthcare solution, promising real‐time patient protection and enhancing the security and durability of long‐term measurement and treatment strategies, representing a significant advancement in the safety and effectiveness of BCI applications in patient care.

## 2 Result

### 2.1 Overview of BrainGuard

For efficient full‐field von Mises strain prediction, we first propose the feature‐based emulator which rationally combines Gaussian Process emulators and model reduction technique to lift the limitations^[^ ^82^, ^83^ ^]^ of traditional Gaussian Process emulators in high‐dimension full‐field biomechanics prediction. The proposed framework utilizes model reduction technique to construct a feature space for efficient low‐rank computation with minimal loss of information.^[^ ^73^, ^74^, ^75^ ^]^ As a data‐driven scheme, it avoids time‐consuming processes such as assembling stiffness matrices and solving a system of equations in high‐fidelity finite element simulations. Additionally, the proposed framework is grounded in Bayesian theory,^[^ ^47^, ^48^ ^]^ which provides a principled mechanism for sustaining long‐term robustness. In this framework, a Gaussian process prior encodes assumptions about brain tissue dynamics, with the kernel function capturing essential physiological dynamics and offering interpretability. As new experimental or simulation observations become available, the posterior distribution is updated, refining both the latent function estimates and the associated noise variance, so that measurement errors and gradual signal drifts are effectively considered and processed in the model. Through Bayesian theory, the likelihood of new data is combined with the prior to derive an updated posterior, which then serves as the prior for subsequent inference. This recurrent observation–update cycle enables the system to adapt to electrode drift, device aging, and patient‐specific variability, aligning the predictive distribution with both new observations and current knowledge and thereby keeping performance robustness over long‐term monitoring.

**Figure 3:**
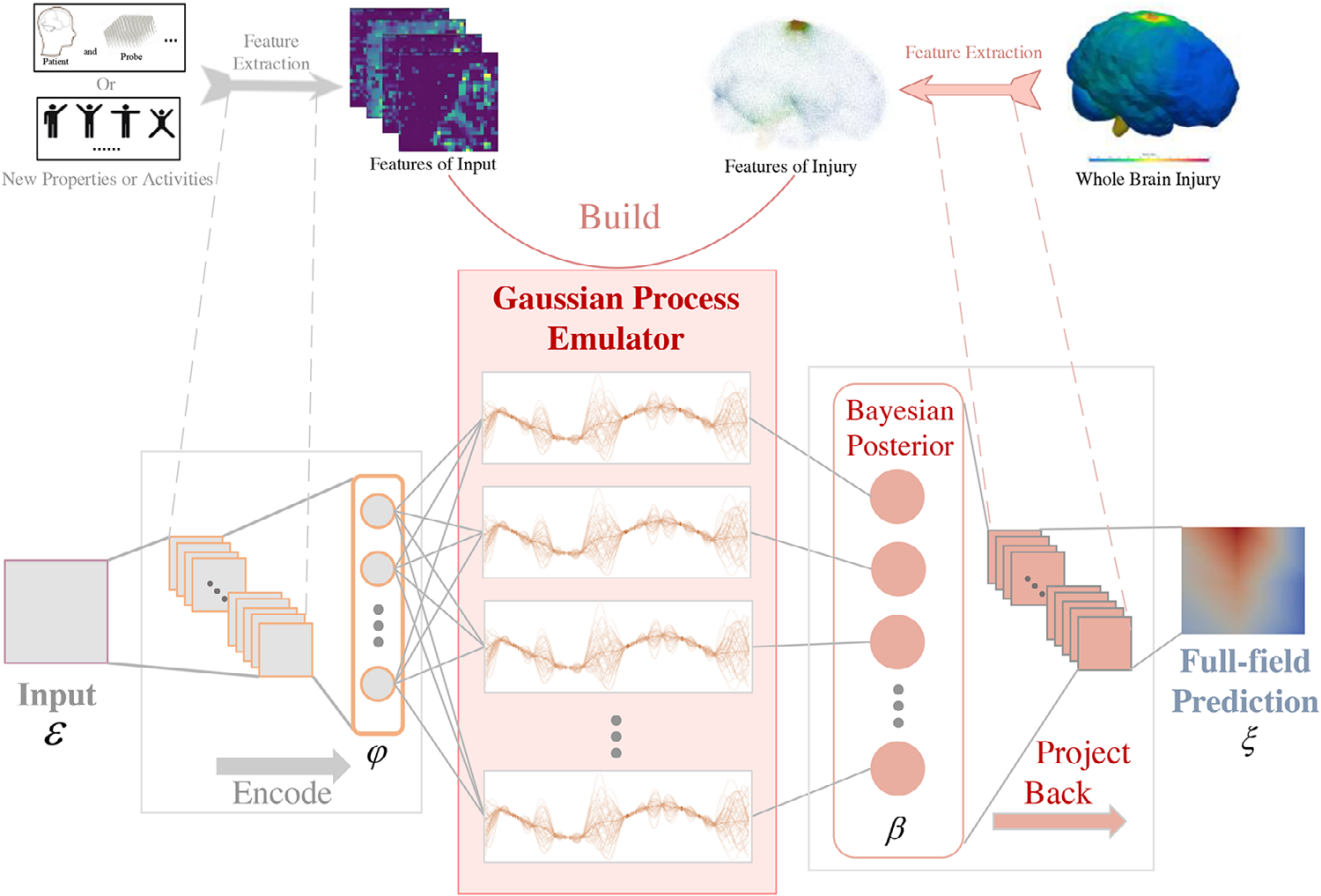
The training and workflow of the feature‐based predictor/monitor. In training phase, the “Encode” (if needed) and “Project Back” phases will learn data compression patterns from feature extractions of the raw input (i.e., patient and BCI properties or daily activities) and raw high‐dimension output (i.e., whole brain injury). Then the features of raw input and raw output are used to build the latent Gaussian process emulator. During training, the Gaussian process emulator naturally evaluates noise within the data and incorporates it into the correlation representation. After finishing the training, given new input, through firstly predicting the features and then project it back to the full output, the emulator can efficiently and accurately emulate the whole brain injury.

The workflow is assisted by a feature capturing scheme mentioned above to capture the mapping of the input $\varepsilon \in R^{I}$ and the features $\beta \in R^{r}$ of output $\xi \in R^{J}$, to enhance the performance of Gaussian process emulator for high‐dimensional predictions, and the workflow in **Figure** **3** of the emulator can be briefly described as follows:

(1) $$\varepsilon \in R^{I}\left(\underset{Encode}{\Rightarrow }\varphi \in R^{K}\right)\underset{Emulate}{\Rightarrow }\beta \in R^{r}\underset{\mathrm{Pr}oject}{\Rightarrow }\xi \in R^{J}$$

“Encode” in formula (1) is flexible, which further provides the proposed framework with the potential for dealing with high‐dimensional input. “Encode” in parentheses will be frozen in this problem to seek for more effective function spaces mapping. For the “Emulate” in the workflow, we build *r* independent Gaussian Processes from the encode space φ (𝜺 in this problem) with each principal component coefficient β_*k*_ (scalar):

(2) $$\beta_{k}∼GP\left(\mu_{k}\left(\varepsilon \right),C_{k}\left(\varepsilon ,\varepsilon^{\prime }\right)\right)\begin{array}{ccc} & for & k=1,\text{…},r \end{array}$$

where µ_*k*_ represents the mean function, *C_k_* is the covariance function. In this process, we can naturally evaluate the inherent noise within the data and incorporate it into the correlation representation of the latent space during training. Finally, we “Project” back to obtain the expectations and variances of the raw high‐dimensional output (the strain field of the whole brain) via

(3) $$E\left[\xi \right]=\mu_{\zeta }+\tilde{\Phi }E\left[\beta \right]$$

where $\tilde{\Phi }$ are called the reduced representation of $\hat{\zeta }$. From a mathematical perspective, the Gaussian process emulator layer in the middle is equivalent to a neural network with infinite width, showing the powerful capability of pattern capture in the whole workflow with limited data.^[^ ^84^, ^85^ ^]^ Meanwhile, it can naturally handle noisy dataset and provide uncertainty estimates, although we focus more on accurate prediction in this task. Actually, this paper proposes a general framework for operator learning based on kernel methods, which establishes a nonlinear mapping between the function space of initial boundary conditions or physical properties and the function space of high‐resolution brain injury. More details in training and prediction are shown in Materials and Methods.

Building on this foundation, BrainGuard focuses on the construction of interdependent offline surrogate models of trustworthy digital brain twins. BrainGuard is tailored for each patient with efficient prediction and real‐time monitoring of patient‐specific whole brain von Mises strain. The von Mises strain represents the effective shear strain of the brain tissue and has been proven to have a sensitive correlation with brain tissue injury and death.^[^ ^24^, ^27^, ^50^, ^51^ ^]^ to comprehensively quantify brain injury. The metrics for quantifying brain injury are flexible and can be adjusted based on the patient‐specific medical needs.

Due to ethical constraints and privacy concerns, collecting human experimental data to train and validate *BrainGuard* presents substantial challenges. The studies involving BCI‐induced brain injuries are subject to rigorous ethical review processes and require informed consent, with the associated risks significantly limiting patient participation. These ethical, legal, and administrative barriers collectively hinder the acquisition of sufficient high‐quality, real‐world data. Despite extensive efforts to identify publicly available datasets on BCI‐induced brain injuries, including both experimental and simulated data, no existing resource was found to comprehensively fulfill the requirements of this study—namely, datasets that fully characterize and quantify whole‐brain injuries caused by BCI interventions.

To address these challenges, ongoing efforts include physical experiments conducted, which are currently progressing but remain under ethical review. In parallel, comparative analyses with relevant publicly available datasets and the expansion of validation components will be presented to enhance the reliability of the framework.

Building on these efforts, this study emphasizes enhancing model complexity to improve the fidelity of brain injury modeling, building upon prior research.^[^ ^24^, ^25^, ^26^, ^27^ ^]^ By incorporating detailed material properties, geometric features, and mechanical behaviors, we aim to capture the nuanced dynamics of BCI–brain interactions within a high‐resolution simulation framework. Looking ahead, we plan to pursue interdisciplinary collaborations with hospitals and neuroscientists to advance this research. Our initial objective is to collect human experimental data from volunteers, thereby reducing reliance solely on finite element (FE) simulation. Subsequently, we will integrate both experimental and simulated data into a unified dataset to train BrainGuard, enabling the model to leverage both real‐world and high‐fidelity synthetic information for improved generalizability and precision.

Furthermore, collaboration with neuroscientists will enhance our understanding of critical brain injury mechanisms, including neuroinflammatory responses and alterations in neural connectivity following BCI implantation. These insights will contribute to the development of more robust and physiologically informed brain injury quantification methodologies from an interdisciplinary perspective. Ultimately, we envision that these efforts will significantly strengthen BrainGuard's capacity to assess and monitor brain injuries in clinical settings, thereby improving its applicability and impact in patient care.

**Figure 4:**
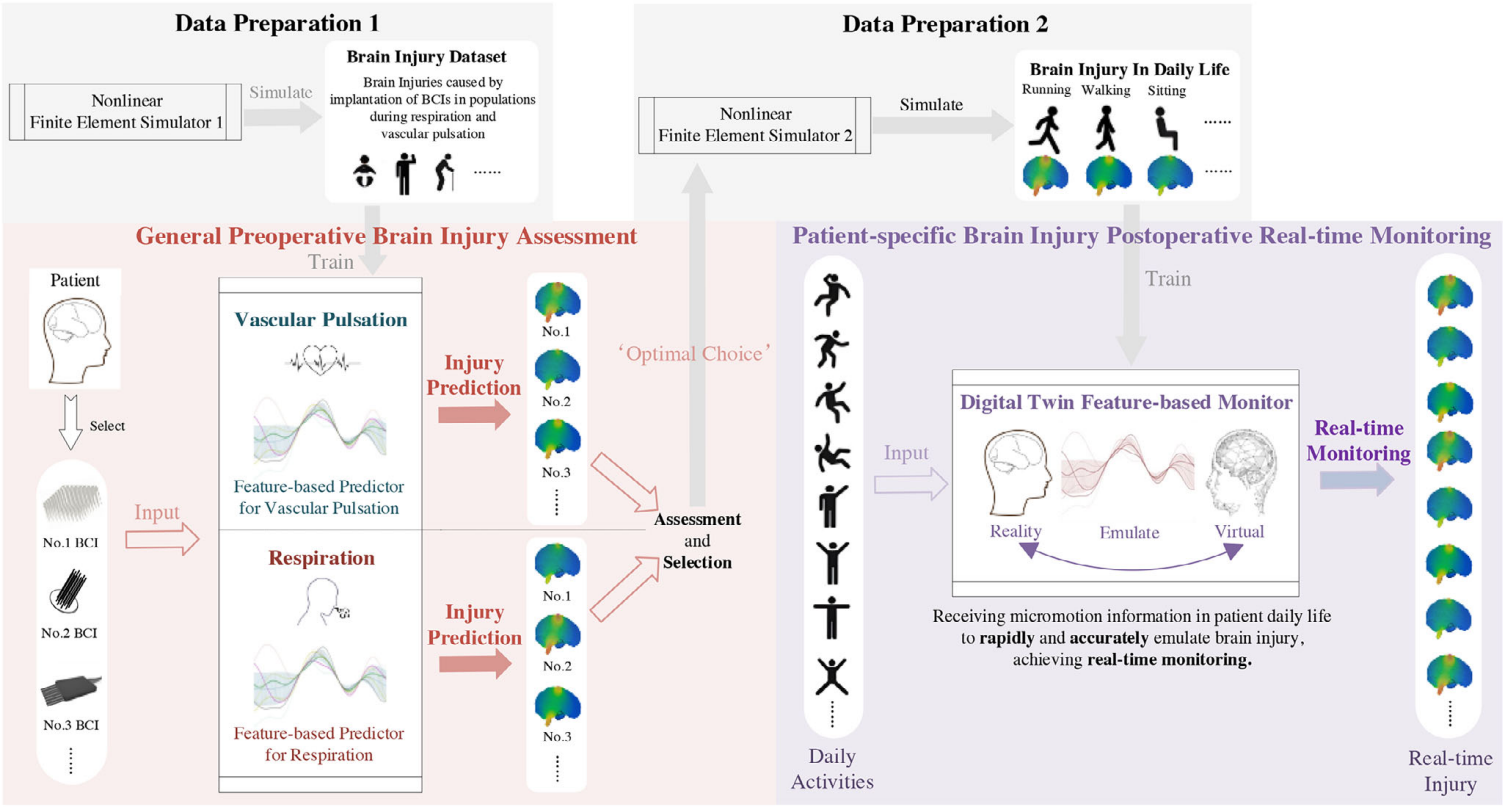
The workflow of BrainGuard. First, in general preoperative brain injury assessment module, before the start, nonlinear finite element 1 generate the high‐fidelity dataset described the brain injuries caused by different implanted BCI in populations during respiration and vascular pulsation, to train feature‐based predictors. In injury prediction phase, BrainGuard receive the mechanical properties of patients and BCI to efficiently predict whole brain injuries caused by BCI for patients. Then in assessment and selection phase, BrainGuard assess each injury prediction to get the “Optimal Choice”. The “Optimal Choice” will be transmitted to data generation 2 to drive nonlinear finite element 2 which simulate the patient‐specific brain injury in daily life, to train the feature‐based monitor which provide the real‐time monitoring. In real‐time monitoring phase, BrainGuard, can real‐time emulate the patient‐specific brain injury through receive the intensive daily activity information. It is worth noting that this framework can naturally evaluate and process different levels of noise hidden in the data, which enhances the long‐term robustness and reliability of the system. We utilized several BCI,^[^ ^60^, ^61^ ^]^ brain injury simulations^[^ ^62^ ^]^ in order to better present the workflow.

BrainGuard (**Figure** **4**) consists of two functional modules: 1) general preoperative brain injury prediction and 2) patient‐specific postoperative real‐time monitoring. The *preoperative prediction* module primarily focuses on forecasting brain injuries before implantation, supplemented by an injury assessment and device selection phase. Prior to clinical use, the nonlinear finite element simulator (Simulator 1) is used to generate a high‐fidelity dataset that statistically models BCI‐induced brain injuries under various conditions^[^ ^53^, ^54^, ^55^, ^56^ ^]^ and use it to train predictors to ensure the accuracy and reliability. In the *injury prediction phase*, two feature‐based predictors—trained on the aforementioned high‐fidelity dataset—take patient and BCI properties as inputs to estimate brain injury caused by respiration and vascular pulsation, respectively.^[^ ^14^, ^29^ ^]^ Respiration and vascular pulsation are recognized as the primary physiological processes driving brain micromotion.^[^ ^22^, ^57^, ^64^ ^]^ During the assessment and selection phase, the model identifies the BCI design that minimizes brain injury for the patient among multiple candidates.^[^ ^57^ ^]^ The properties of this optimal BCI are recorded alongside the patient's brain characteristics to establish an individualized “optimal choice.” This information is subsequently passed to the postoperative monitoring module, thereby ensuring that patients receive personalized treatment strategies that effectively reduce injury risks and associated complications.

The *patient‐specific real‐time postoperative monitoring module* aims to provide efficient brain injury real‐time monitoring. Before the start, the recorded “optimal choice” will be received in data preparation 2, in order to drive the nonlinear finite element simulator 2 to generate datasets which accurately and densely simulate the patient‐specific brain injury due to brain micromotion in daily life, serving as the abundant raw material for training to guarantee high quality real‐time monitoring. The trained feature‐based monitor is also built by the Gaussian Process emulator and model reduction technique. In real‐time monitoring, the feature‐based monitor is provided to real‐time monitor the patient‐specific injury of the brain by receiving displacement data from displacement sensors in BCI,^[^ ^58^, ^59^ ^]^ such as DVRT, to provide doctors and patients with reliable and real‐time full‐field von Mises strain to reduce the risks of brain injury and the failure of high‐precision measurement of BCI in postoperative rehabilitation and daily life, offering digital assurance for long‐term treatment strategies and even more challenging neuroscience researches. Representative results, including statistical analyses and comparisons under different conditions, will be presented later, while more comprehensive cross‐sectional views will be provided in the Supplementary Information S1.

It is worth noting that this framework can naturally evaluate and process different levels of noise hidden in the data, which enhances the long‐term robustness and reliability of the system. In this study, we propose a practical yet straightforward approach for implementing a digital twin brain, which utilizes sensor‐transmitted relative displacement data to predict full‐field, patient‐specific brain injury. This approach is grounded in the understanding that BCI‐induced brain injury is primarily driven by the relative motion between neural tissue and the implanted probe. As such, relative displacement serves as a direct and integrative variable that encapsulates the combined effects of key physiological factors, including body movement, mechanical input, and cerebral pulsation. While this method may not capture all physiological indicators, it offers a reliable and efficient means of quantifying injury risk. Collecting displacement data at high temporal resolution further enhances the model's ability to characterize the dynamic interactions between the brain and the BCI, albeit without achieving perfect accuracy. Furthermore, we are actively pursuing collaborations with hospitals to establish brain injury datasets enriched with diverse physiological indicators through advanced sensing technologies. These efforts aim to improve the fidelity of surrogate models for digital twin brains and to advance our understanding of the underlying mechanisms and progression of BCI‐induced brain injuries.

The metric von Mises strain that we choose to quantify BCI caused brain injury has been more widely applied and researched compared to others due to its strong association with local brain bleeding, the formation of cell sheaths and significant brain tissue loss,^[^ ^24^, ^27^, ^50^, ^51^ ^]^ which will unpredictably lead to neurological disorders, paralysis, and even death.^[^ ^18^, ^19^, ^20^ ^]^ To better demonstrate the results, we normalize the full field von Mises strains. The relative error of the predicted injury to the reference injury for each point is calculated by:

(4) $$error=\frac{S_{pre}}{S_{pre,\max}}-\frac{S_{ref}}{S_{ref,\max}}$$

where *S_pre_* denotes predicted von Mises strains in one point, *S*_*pre*,max _ is the max predicted von Mises strain in the whole brain, *S_ref_* denotes reference von Mises strain in one point, and *S*_*ref*,max _ is the max reference von Mises strain in the whole brain.

### 2.2 General Preoperative Brain Injury Prediction

Let us evaluate the performance of two feature‐based predictors for quantifying the injury of different BCI to the patient brains during representative physiological processes in human. Two predictors capture mappings from high‐fidelity datasets generated by nonlinear finite element simulator 1 with different initial settings, respectively, for preoperative prediction of the von Mises strain in the whole brain caused by the brain micromotion during respiration^[^ ^63^, ^64^ ^]^ and vascular pulsation.^[^ ^14^, ^65^ ^]^ It is worth emphasizing that the proposed framework achieves material generalization by leveraging a high‐fidelity dataset constructed through comprehensive and physically accurate modeling. Moreover, the framework—built upon Gaussian processes, which have been rigorously validated both theoretically and practically across diverse engineering domains^[^ ^35^, ^49^, ^84^ ^]^ —is capable of accurately predicting brain injuries caused by BCI with varying geometric configurations through retraining. These efforts aim to further improve predictive accuracy across diverse tasks while reducing the reliance on repeatedly generating costly high‐fidelity datasets.

**Figure 5:**
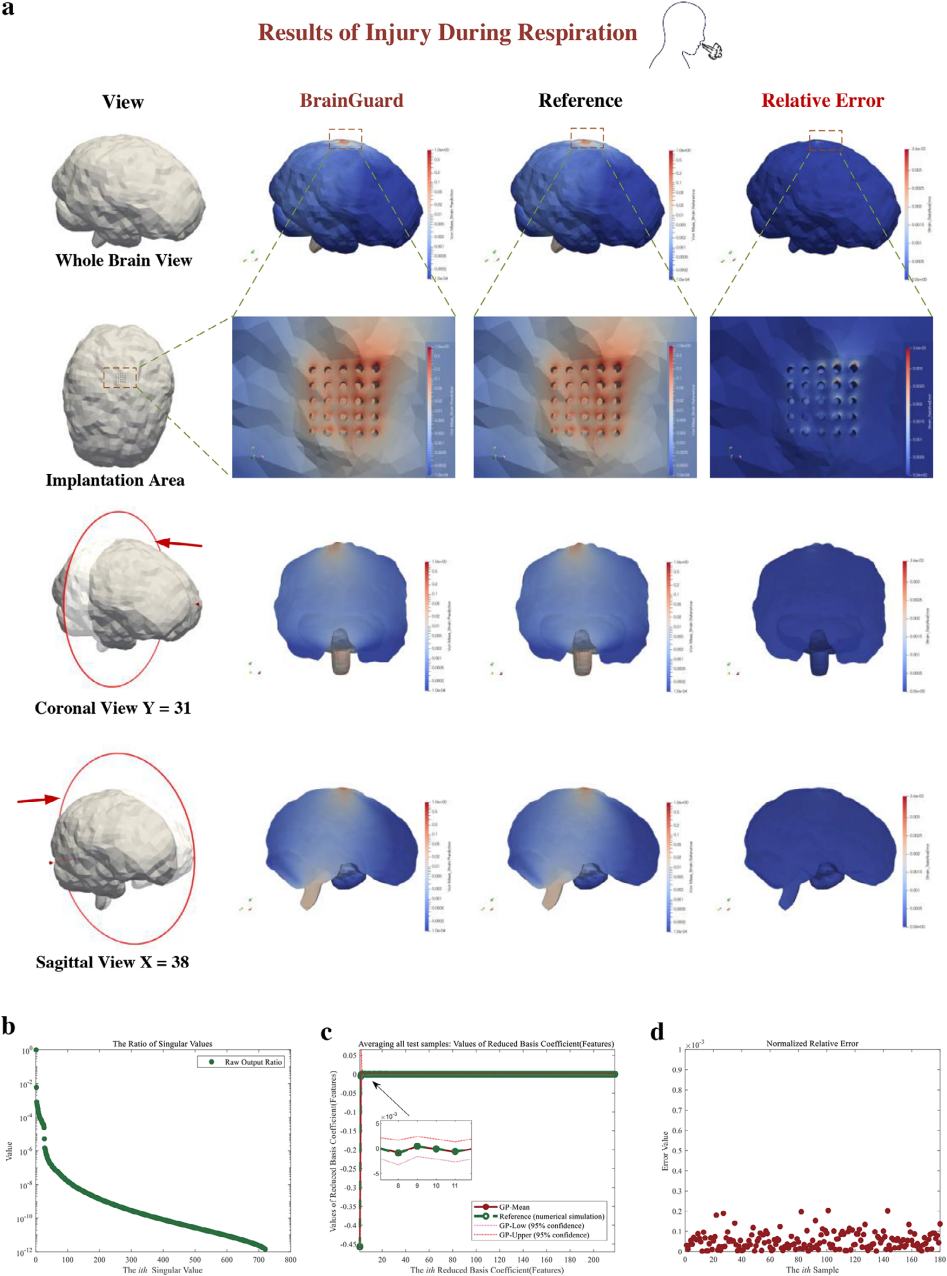
Result of prediction of brain injury caused by respiration using feature‐based predictor. a) Prediction, reference, and relative error of whole brain injury (von Mises strain) from a whole brain view, an enlarged view of the implantation area, and coronal view and sagittal view. b) Each ratio of the singular value of the output in prediction. The ratio is the proportion of the square of singular value of output to the sum of squares of singular values of output. c) The prediction (Red solid point) and reference (Green hollow point) of the features of the output (von Mises strain field) average all test samples. The confidence interval is given in enlarged graph. d) The normalized relative error of each sample averaging all measurement points.

Figure 5 shows the excellent performance of the predictor of emulate the brain injury of respiratory induced brain micromotion. The predicted results of the whole brain von Mises strain can be found in **Figure** **5**a. The comparison of the predicted strains and the reference strains averaging all testing samples is illustrated in Figure 5a. The relative error is mostly less than 10^−4^ while in the implanted area, the error does not exceed 4 × 10^−3^. This result illustrates that the proposed predictor can provide accurate prediction of the whole brain injury of respiratory induced brain micromotion.

In order to further explore the reasons why the feature‐based predictor can provide such accurate predictions, Figure 5b‐d depicts the performance of the predictor in the sub phases. One of the most important sub phases of the feature‐based predictor is to capture features of high‐dimensional output, i.e., whole brain von Mises strain, preserving it in the feature space while minimizing information loss. In order to clearly describe capturing features, in Figure 5b, we present the information ratio of each feature, which is the proportion of the square of singular value of output to the sum of squares of singular values of output, sorted in descending order. The minimum ratio of features we retain is less than 10^−11^, indicating that the effectiveness of capturing features of the proposed predictor, which represents minimal information loss when compressing high‐dimensional whole brain injury. After compressing the whole brain injury into the feature space, we only need to establish Gaussian processes for raw input and each feature of raw output, separately, which greatly reduces the resource consumption of the predictor for whole brain prediction. Figure 5c shows the average value of each predicted and reference feature; within the predicted confidence interval, the reference and prediction are almost identical. Subsequently, we evaluated the accuracy of individual samples to demonstrate our ability to provide general prediction for different patients. In Figure 5c, the relative error of each sample averaging the whole brain measurement points and the relative error of each sample is presented. The subsequent Table 1 shows the efficiency of the feature‐based predictor, which is only 1/1600 compared of the numerical simulation time.

**Figure 6:**
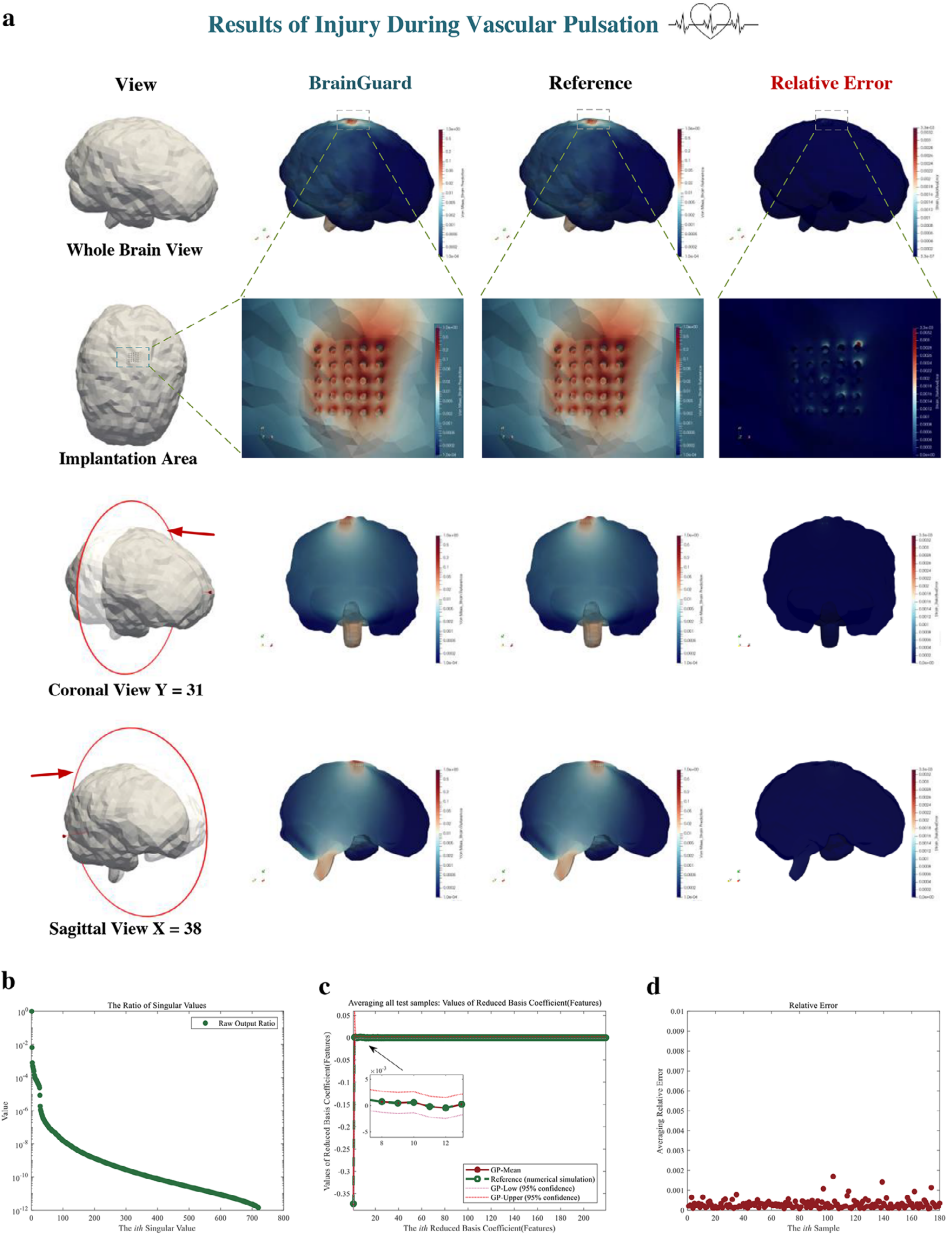
Result of prediction of brain injury caused by vascular pulsation using feature‐based predictor. **a)** Prediction, reference, and relative error of whole brain injury (von Mises strain) from a whole brain view, an enlarged view of the implantation area, and coronal view, and sagittal view. b) Each ratio of the singular value of the output in prediction. The ratio is the proportion of the square of singular value of output to the sum of squares of singular values of output. c) The prediction (Red solid point) and reference (Green hollow point) of the features of the output (von Mises strain field) average all test samples. The confidence interval is given in enlarged graph. d) The normalized relative error of each sample averaging all measurement points.

Next, we evaluate the performance of the predictor for brain injury under vascular pulsation induced brain micromotion and the results are presented in **Figure** **6**. To ensure a comprehensive representation of the predictor performance under vascular pulsation, Figure 6a illustrates the comparison of the predicted whole brain von Mises strain and the reference von Mises strain, and the relative error in whole brain, with a whole brain view, an enlarged view of the implantation area, and coronal view and sagittal view. Once again, a strong resemblance between the predicted and reference von Mises strain in the whole brain can be observed, indicating a high level of agreement. We further investigate the relative error and observe the maximum error is less than 4 × 10^−3^.

**Table 1:** Time spent to calculate the full field strain by predictor and simulator under respiration.

	Time [s]
Predictor	Prediction phase
	Draw features	Predict features	Project back
	0.009	0.21	0.001
Simulator	358[22, 63, 64]
Ratio (Simulator / Predictor)		1627.27	

Figure 6b presents each ratio of the singular values of the high‐dimensional full‐field von Mises strain which represents the brain injury under vascular pulsation; the smallest ratio is less than 10^−12^, which suggests that almost all information of the high‐dimension brain injury is retained in the feature space we constructed. To further demonstrate accurate predictive performance under vascular pulsation, we compared the mean and confidence intervals of the predicted features of whole brain injury with the references in Figure 6c; they almost overlap, and the confidence intervals are significantly narrow, indicating the predictor can provide accurate brain injury predictions under vascular pulsation with very strong confidence. Finally, we evaluated the accuracy of individual cases to demonstrate our ability to provide general predictions. We show the relative errors, which quantify the accuracy of predicting the brain injury in individual samples, in Figure 6d. The computational cost of the predictor is only 1/1600 of that of numerical simulation as shown in **Table** **2**. This excellent performance demonstrates that the feature‐based predictor enables to provide general and reliable quantitative references of the brain injury during representative physiological processes for the diagnosis and treatment decisions.

**Table 2:** Time to calculate the full field strain by predictor and simulator under vascular pulsation.

	Time (s)
Predictor	Prediction phase
	Draw features	Predict features	Project back
	0.009	0.21	0.001
Simulator	358[22, 63, 64]
Ratio (Simulator / Predictor)		1627.27	

### 2.3 Patient‐Specific Brain Injury Real‐Time Postoperative Monitoring

We next evaluate the performance of the real‐time monitor, which is based on Gaussian Process regression and used to predict the whole brain von Mises strain in different brain micromotions to quantify the brain injury in daily life. To further simulate real‐world challenges, we consider the robustness of signal transmission over the long‐term use of BCI systems.^[^ ^86^, ^87^, ^88^ ^]^ Specifically, signal transmission errors are modeled as Gaussian noise added to the raw high‐fidelity dataset. The framework is tested under five conditions: no noise, zero‐mean Gaussian noise with SNRs of 10 and 5 dB, and Gaussian noise with non‐zero means (0.001 and 0.005) at 5 dB SNR. The zero‐mean conditions simulate random fluctuations typically assumed in classical models, while the non‐zero mean conditions represent cumulative bias and drift that inevitably arise from electrode instability, environmental variation, or device aging.^[^ ^91^, ^92^, ^93^, ^94^ ^]^ Importantly, introducing non‐zero mean noise at SNR of 5 dB creates a challenging test scenario, as it combines systematic offset with notable signal degradation. By considering both random and biased noise scenarios, these experiments provide a comprehensive validation to evaluate the long‐term robustness of the proposed system.^[^ ^95^, ^96^, ^97^ ^]^

#### 2.3.1 Baseline Performance under Noise‐Free Condition

**Figure 7:**
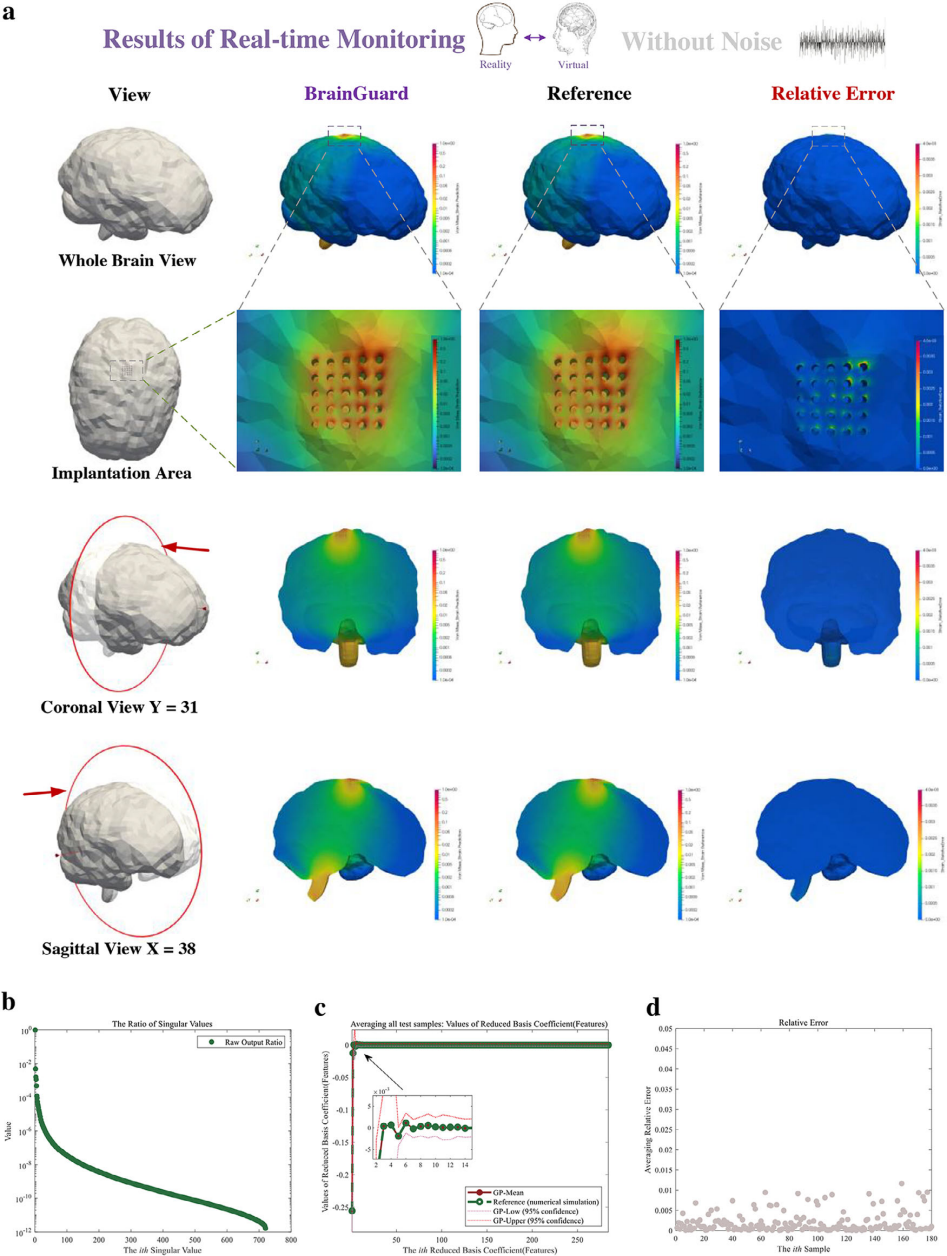
Result of real‐time monitoring of brain injury in daily activity using feature‐based monitor without noise. a) Prediction, reference, and relative error of brain injury (von Mises strain) from a whole brain view, an enlarged view of the implantation area, and coronal view and sagittal view. b) Each ratio of the singular value of the output in prediction. The ratio is the proportion of the square of singular value of output to the sum of squares of singular values of output. c) The prediction (Red solid point) and reference (Green hollow point) of the features of the output (von Mises strain field) average all test samples. The confidence interval is given in enlarged graph. d) The normalized relative error of each sample averaging all measurement points.

We first validate the framework's monitoring accuracy under noise‐free conditions, providing a reference for evaluating robustness in more realistic clinical scenarios. In **Figure** **7**, We first validate the performance of the monitor without noise. We compare the predicted and the reference von Mises strain with the relative error of whole brain, as shown in a whole brain view, an enlarged view of the implantation area, coronal view and sagittal view presented in Figure 7a. A significant level of agreement is evident between the predicted von Mises strain and the reference von Mises strain in all 4 views. To further validate the accuracy of our prediction, we present the relative error on the far right in Figure 7a. In the whole brain prediction, the parts with the largest errors are mainly distributed in the areas where brain tissue contacts the probes and the bottom of the brain. Nonetheless, the maximum error of all measurement points does not exceed 4 × 10^−3^.

Figure 7b illustrates each ratio of the feature. The minimum ratio is less than 10^−12^, suggesting the model reduction technique effectively captures the underlying characteristics of high‐dimensional whole brain von Mises strains in monitoring. As depicted in Figure 7c, the Gaussian Process emulator predicted features exhibit a remarkable consistency with the reference features, which strengthens our confidence in the monitoring ability for the whole brain injury. We finally evaluated the accuracy of the predictions in each sample. The relative error of each testing sample is presented in Figure 7d with errors smaller than 1.5 × 10^−2^ indicating the proposed predictor provides accurate predictions in real‐time.

#### 2.3.2 Robustness to Stochastic Disturbances

**Figure 8:**
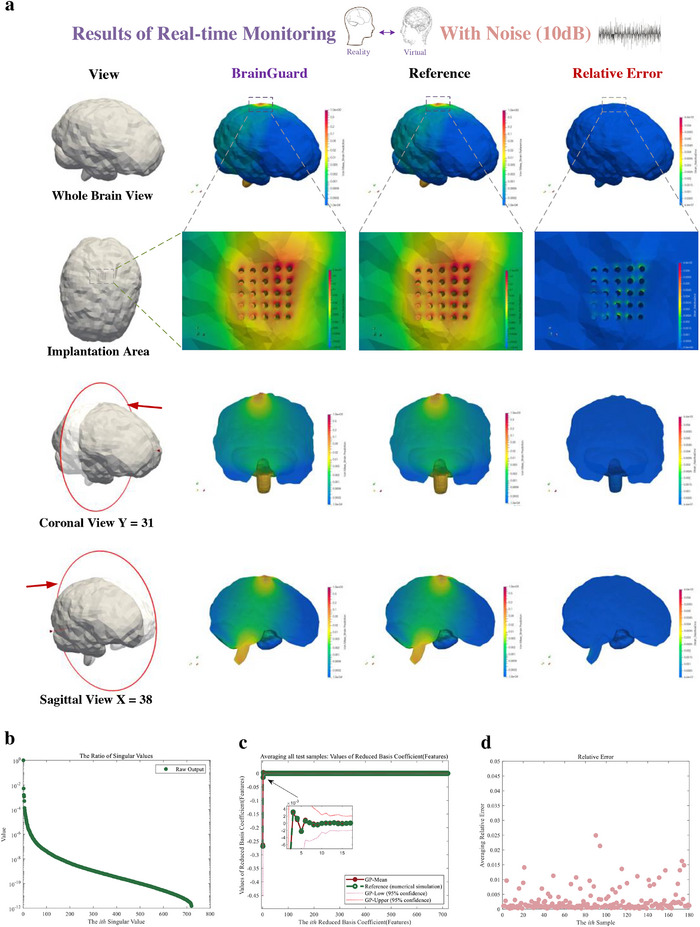
Result of real‐time monitoring of brain injury in daily activity using feature‐based monitor with low‐noise (signal to noise ratio of 10 dB). a) Prediction, reference, and relative error of brain injury (von Mises strain) from a whole brain view, an enlarged view of the implantation area, and coronal view, and sagittal view. b) Each ratio of the singular value of the output in prediction. The ratio is the proportion of the square of singular value of output to the sum of squares of singular values of output. c) The prediction (Red solid point) and reference (Green hollow point) of the features of the output (von Mises strain field) average all test samples. The confidence interval is given in enlarged graph. d) The normalized relative error of each sample averaging all measurement points.

**Figure 9:**
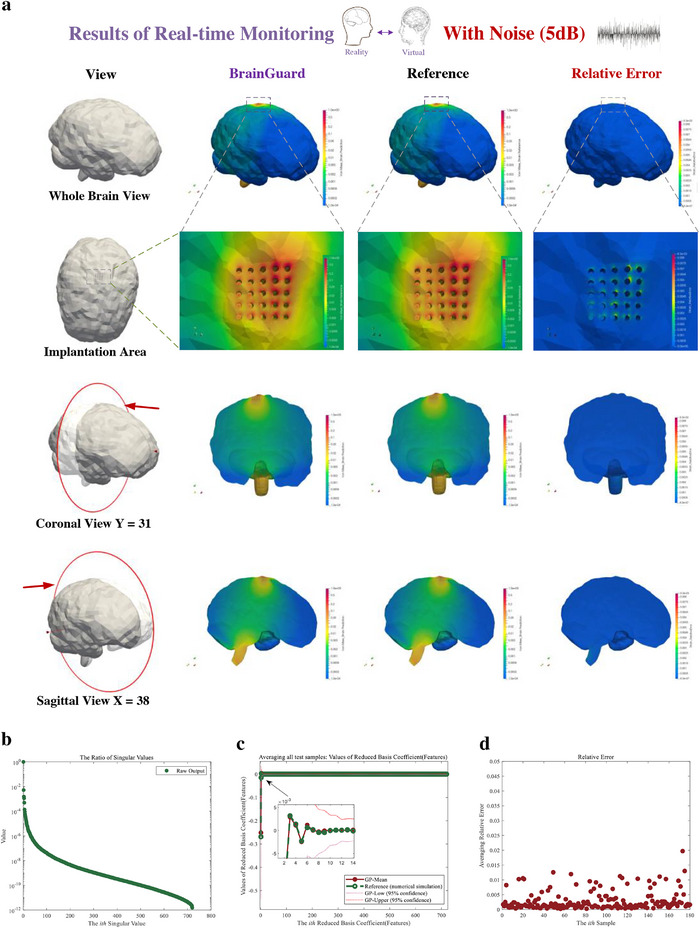
Result of real‐time monitoring of brain injury in daily activity using feature‐based monitor with high‐noise (signal to noise ratio of 5 dB). a) Prediction, reference, and relative error of brain injury (von Mises strain) from a whole brain view, an enlarged view of the implantation area, and coronal view and sagittal view. b) Each ratio of the singular value of the output in prediction. The ratio is the proportion of the square of singular value of output to the sum of squares of singular values of output. c) The prediction (Red solid point) and reference (Green hollow point) of the features of the output (von Mises strain field) average all test samples. The confidence interval is given in enlarged graph. d) The normalized relative error of each sample averaging all measurement points.

**Figure 10:**
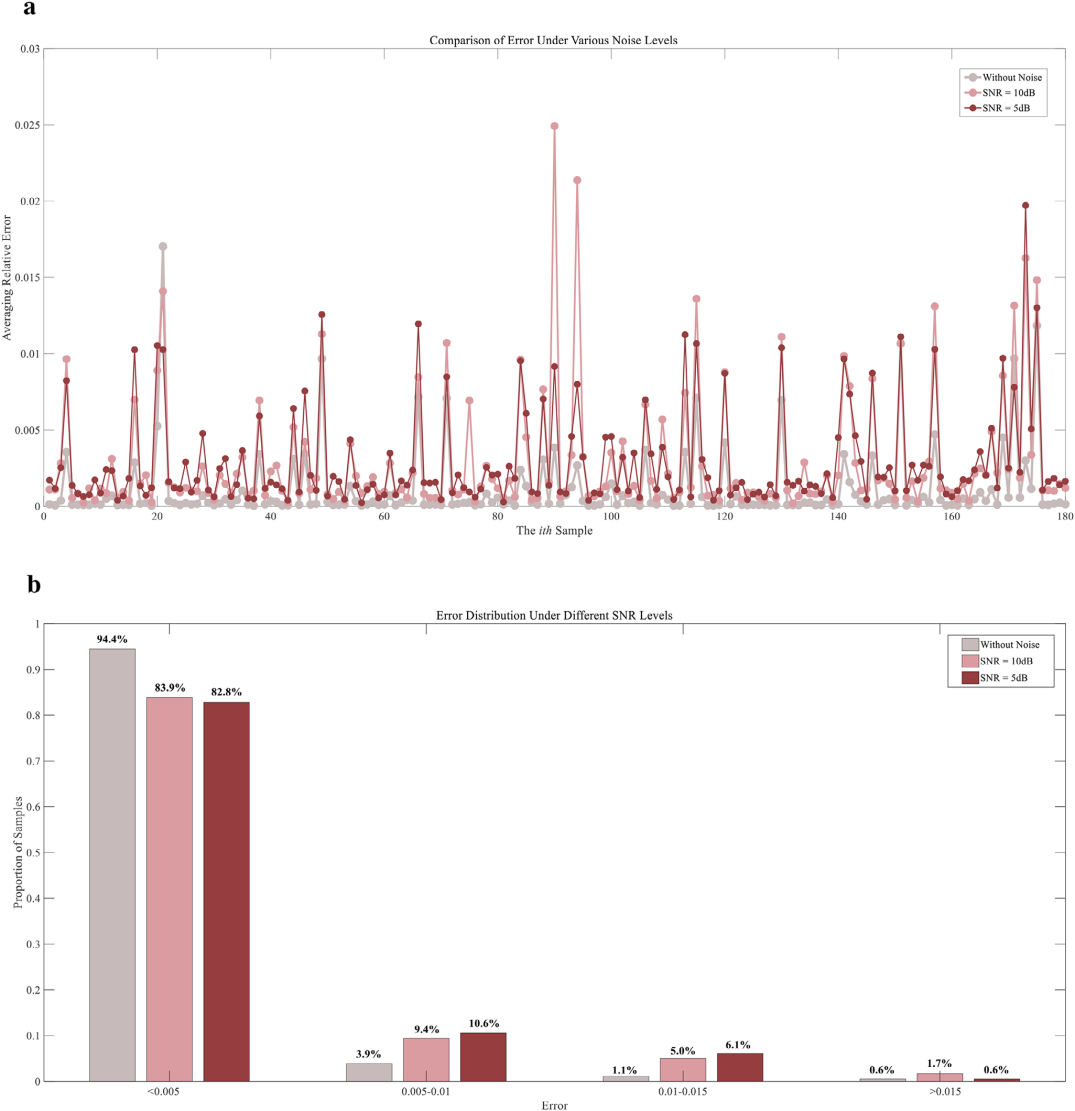
Error analysis of the feature‐based monitor under different SNR levels. a) Comparison of relative error across samples without noise, with 10 dB noise, and with 5 dB noise. b) Error distribution statistics under different SNR levels, showing the proportion of samples falling within given error ranges. The bar colors correspond to the error plots (d) in Figures. 7, 8, 9, allowing direct comparison across conditions.

To validate the robustness against stochastic disturbances that commonly occur in long‐term neural recordings, we then assess the framework under zero‐mean Gaussian noise at different SNRs, as summarized in **Figures** **8** to 10. In Figure 8, we assess its performance under noise environment (SNR of 10 dB). Under low noise disturbance, the predicted and reference von Mises strains show high consistency across the whole‐brain view, the enlarged implantation area, coronal, and sagittal views (Figure 8a). The relative error, also shown in Figure 8a, is still mainly distributed around the probe‐tissue interface, with maximum error below 7 × 10^−3^.

Figure 8b presents the feature ratios, where the minimum value falls below 10^−12^. It demonstrates that even with noise, the model reduction method still effectively preserves essential features of whole‐brain strain fields. As shown in Figure 8c, the Gaussian process emulators accurately predict the reference features with evaluation of noise within the dataset. Finally, the prediction error across test samples (Figure 8d) remains below 2.25 × 10^−2^, confirming high accuracy of the monitor for real‐time emulations. In low‐noise environment, we can observe that the error of each test sample increases slightly compared to without noise, but it still performs outstanding performance for prediction (maximum error below 2.25 × 10^−2^). The proposed framework effectively evaluates and adapts to potential low‐level noise within data.

In **Figure** **9**, we validate the monitor's performance under a challenging noise condition (signal‐to‐noise of 5 dB). Despite strong noise interference, the predicted whole‐brain von Mises strain remains in strong agreement with the reference across all views: whole‐brain, implantation area, coronal, and sagittal (Figure 9a). Moreover, relative error analysis reveals that the primary discrepancies occur near the probe‐brain interface and the base of the brain, with the maximum error staying below 9 × 10^−3^, demonstrating robust predictive performance of the proposed framework under high noise. Figure 9b shows that the minimum feature ratio remains below 10^−12^, indicating that the model reduction approach effectively captures the essential characteristics of the high‐dimensional strain field. In Figure 9c, the predicted features of the Gaussian process emulators closely match the reference, confirmed the effectiveness of establishing mappings in the reduced and feature space under high noise. Finally, the relative error of each testing sample, shown in Figure 9d, remains below 2 × 10^−2^. The lower relative error presents that the proposed framework can accurately predict whole brain injury through natural noise assessment even in high noise levels (after long‐term use).

**Figure** **10** provides a comprehensive assessment of prediction errors under different SNR levels. As shown in Figure 10a, the introduction of noise increases the fluctuation amplitude of sample‐wise relative errors, with 10 dB conditions remaining close to the noise‐free baseline and 5 dB conditions producing more spikes. Figure 10b presents the error distribution across all samples: over 94% of cases stay below 0.005 without noise, 83.9% under 10 dB and 82.3% under 5 dB, with only a small proportion of samples exceeding 0.015. These results demonstrate that even under severe zero‐mean noise, the framework preserves reliable accuracy for the majority of samples, with slight degradation limited to the most challenging conditions that the magnitude of the fluctuating noise surpasses that of the input. This robustness arises from the adaptive noise estimation and processing in Gaussian process regression, which considers and models inherent noises for posterior inference with uncertainty. Overall, the analysis shows that the framework maintains stable performance under zero‐mean Gaussian noise, whereas more challenging scenarios with systematic bias will be examined in subsequent sections.

#### 2.3.3 Robustness to Bias and Drift with Strong Disturbance

**Figure 11:**
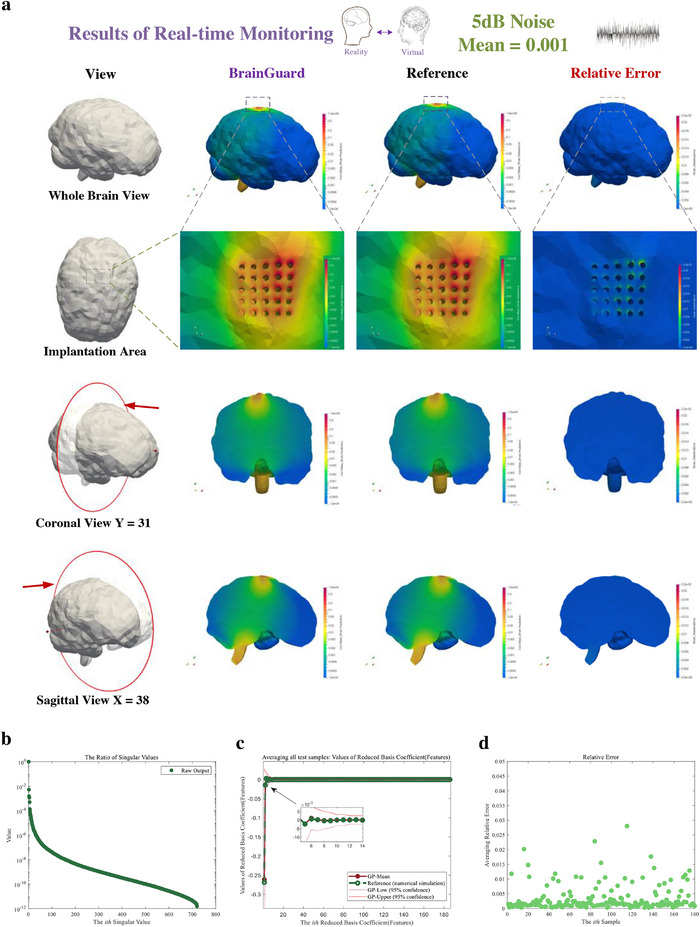
Result of real‐time monitoring of brain injury in daily activity using feature‐based monitor under biased noise (SNR = 5 dB, mean = 0.001). a) Prediction, reference, and relative error of brain injury (von Mises strain) from a whole brain view, an enlarged view of the implantation area, and coronal and sagittal views. b) Ratio of the singular values of the predicted output. c) Predicted (red solid points) and reference (green hollow points) features averaged across all test samples with confidence interval. d) Normalized relative error of each sample averaged across all measurement points.

Finally, to further evaluate long‐term robustness of BrainGuard, we consider non‐zero mean Gaussian noise at 5 dB, representing the combined influence of bias, drift, and strong disturbance from electrode instability, environmental factors, and device aging. **Figure** **11** evaluates the framework under biased noise with a mean of 0.001 at 5 dB SNR. The predicted von Mises strain agrees well with the reference across whole‐brain and sectional views (Figure 11a) with a maximum error reaches 2.2 × 10^−2^. The singular value spectrum in Figure 11b shows rapid decay, with the smallest ratio below 10^−12^, confirming that reduced‐order modes preserve dominant strain dynamics. In Figure 11c, predicted features closely track the reference with narrow confidence intervals, suggesting reliable feature‐space mapping despite systematic bias. Figure 11d shows most sample‐wise errors below 0.02, with only scattered deviations. This indicates that although non‐zero mean noise introduces residual offsets, the framework based on Gaussian process mitigates their impact through adaptive variance modeling.

**Figure 12:**
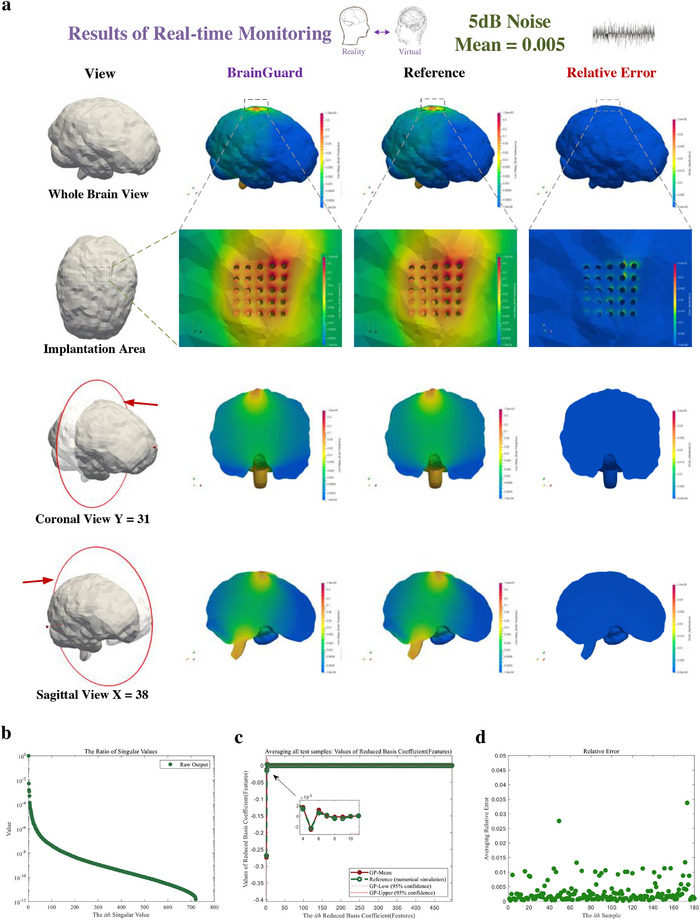
Result of real‐time monitoring of brain injury in daily activity using feature‐based monitor under stronger biased noise (SNR = 5 dB, mean = 0.005). a) Prediction, reference, and relative error of brain injury (von Mises strain) from a whole brain view, an enlarged view of the implantation area, and coronal and sagittal views. b) Ratio of the singular values of the predicted output. c) Predicted (red solid points) and reference (green hollow points) features averaged across all test samples with confidence interval. d) Normalized relative error of each sample averaged across all measurement points.

**Figure** **12** examines biased noise with a mean of 0.005 at 5 dB SNR. The predicted strain generally follows the reference (Figure 12a) with the maximum error increases to 3.6 × 10^−2^, reflecting stronger distortion from cumulative bias. Figure 12b shows the singular value ratios remain stable across modes though with stronger disturbance, confirming that the low‐rank basis effectively captures key strain features. In Figure 12c, predicted features align with the reference but exhibit wider confidence intervals, indicating reduced certainty under stronger bias. Figure 12d shows that most errors stay under 0.025, although more samples exceed 0.015 compared with the 0.001 mean case. These results show that stronger bias increases residual errors and shifts them into the predictive mean, but the framework with adaptive noise processing limits this effect and prevents major performance degradation, allowing the model to remain robust.

**Figure 13:**
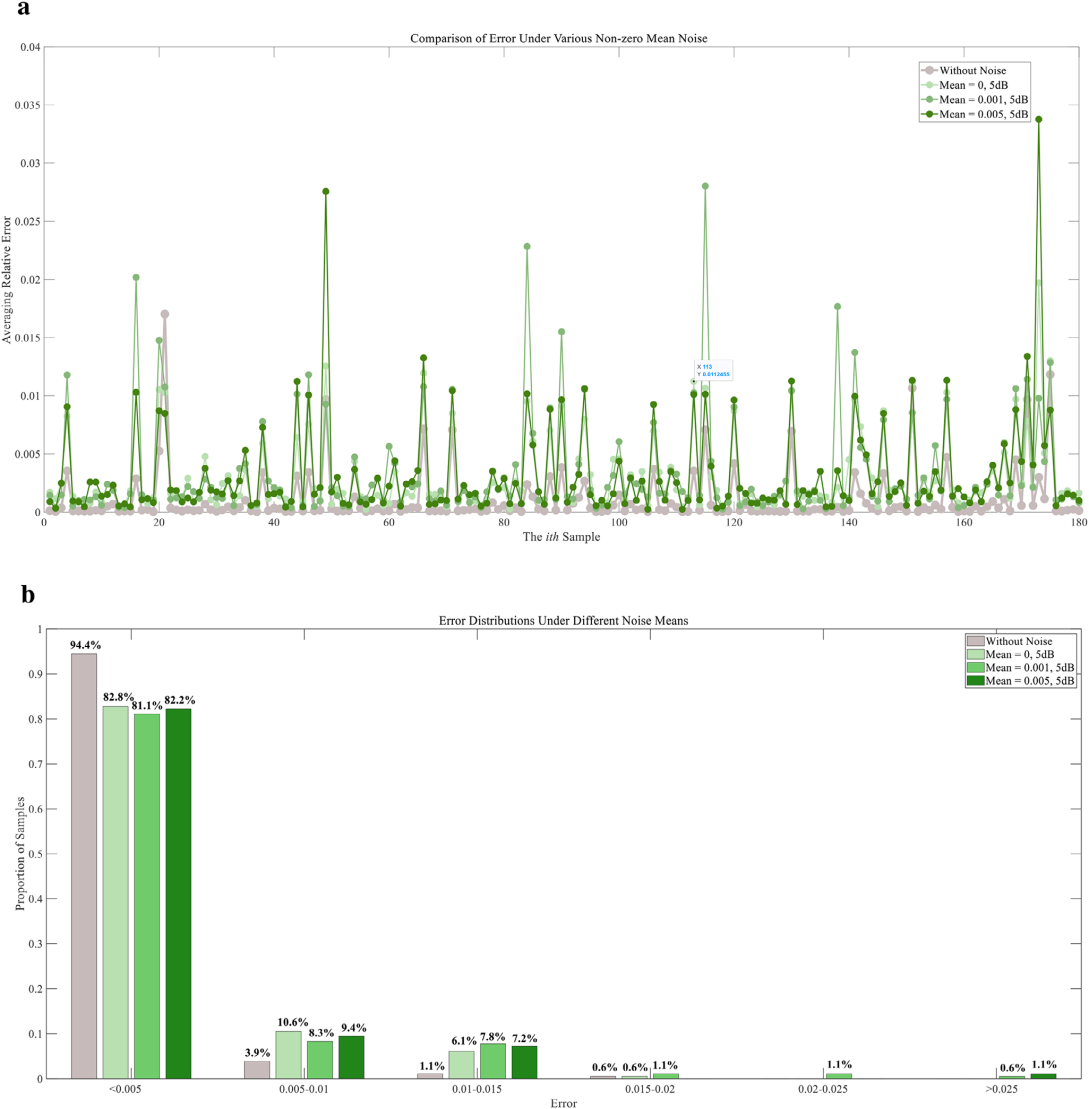
Comparative error analysis across biased noise conditions. a) Sample‐wise relative error under noise‐free, zero‐mean (5 dB), mean = 0.001 (5 dB), and mean = 0.005 (5 dB) conditions. b) Statistical error distributions across conditions, showing proportions of samples within defined error ranges. The bar colors correspond to the error plots (d) in Figures. 7, 10, 11, 12. For clarity of comparison, the color in Figure 10 will be change in this figure.

**Figure** **13** provides a comprehensive and comparative analysis of biased noise effects. As shown in Figure 13a, non‐zero mean noise produces stronger fluctuations than the zero‐mean case, with the largest deviations at mean = 0.005. The statistical distributions in Figure 13b confirm this trend: while most samples remain in the low‐error range, the proportion of higher‐error cases (>0.015) increases as bias grows. This demonstrates that compared to zero‐mean noise, biased noise not only amplifies fluctuations but also introduces a systematic residual component that accumulates across predictions. Even under these adverse conditions, the framework sustains stable performance: in Figure 13b, over 80% of samples remain below 0.005, and only a limited fraction exceed 0.015 even at mean = 0.005. From a theoretical perspective, the reduced‐order representation preserves the main low‐rank modes of the strain field, limiting systematic drift in the core signal. In parallel, the adaptive variance modeling and processing in Gaussian process regression reduces the influence of noisy components. Working together, these two mechanisms allow the framework to handle biased noise effectively, keeping errors limited and system robustness.

#### 2.3.4 Computation Efficiency

In addition to accuracy with robustness, the proposed monitor offers substantial computational advantages compared to traditional methods. The numerical simulations were performed on a Dell OptiPlex 7000 workstation equipped with an Intel Core i9‐12900 CPU (12th Gen, 16 cores, 24 threads, base frequency 2.40 GHz), 64 GB RAM. The computational cost of the predictor is summarized in **Table** **3**, which is 0.22s for each testing sample with more than 3 × 10^5^ measurement points and only 1/2400 of the numerical simulation. The fast and accurate monitoring performance under various noise levels highlights the significant advantages provided by the proposed monitor, which provides an efficient method and basic groundwork for the computational mechanics based medical digital twin. The natural incorporation of noise evaluation within the framework contributes to enhancing the long‐term robustness of the device. Meanwhile, the interpretability of the framework has great potential to establish a solid foundation for guiding medical decision‐making.

**Table 3:** Time spent to calculate the full field strain by real‐time monitor and simulator in daily activity.

	Time [s]
Real‐time monitor	Prediction phase
	Draw features	Predict features	Project back
	0.009	0.21	0.001
Simulator	536[22, 63, 64]
Ratio (Simulator / Real‐time monitor)		2436.36	

## 3 Discussion

We introduce BrainGuard, an innovative and dependable AI platform aimed at constructing trustworthy patient‐specific digital brain twins to efficiently provide reliable and quantitative patient‐specific reference and real‐time monitoring of brain injuries resulting from BCI due to brain micromotion. While the primary focus of this study is on rapidly and accurately predicting brain injuries caused by BCI, the methodology extends beyond this specific application. BrainGuard offers a versatile framework for efficiently predicting adverse reactions to implants (not limited to BCI) and enabling real‐time monitoring to ensure patient safety during daily activities. This framework supports optimal surgical decision‐making to minimize potential patient injuries and provides long‐term, real‐time patient protection post‐surgery.

Our proposed framework demonstrates excellent performance, including rapid and accurate prediction of whole‐brain injuries, effective feature space construction, and mapping of input to features. Due to ethical and privacy constraints, obtaining data from human experiments to train BrainGuard is challenging. To balance accuracy and efficiency, we use simplified patient brain descriptions during data generation. Future efforts may focus on constructing datasets of higher quality by introducing comprehensive material indicators to describe human brains, quantifying geometric differences among brains of different patients, developing and implementing brain injury assessments tailored to individual patients, and collaborating with hospitals to collect human experimental data from volunteers. In actively fostering collaborations with hospitals, we plan to collect a more comprehensive dataset to enable more accurate quantification of brain injury and the development of digital twin brain models. Simultaneously, we are actively exploring extensions and generalizations of the proposed framework to broaden its applicability across a wider variety of BCI. In particular, we are investigating Gaussian process‐based transfer learning methods, aiming to efficiently adapt our trained emulator to new or less‐studied BCI. By transferring biomechanics knowledge acquired from common electrode simulations to specific or emerging cases, this approach enables rapid adaptation with reduced data and computational costs while maintaining high prediction accuracy.

Integrating BrainGuard with treatment strategies offers great potential to deliver comprehensive and reliable patient care throughout the entire illness‐to‐recovery process, representing a promising direction for future research and clinical application.

## 4 Materials and Methods

### Nonlinear Finite Element Simulator for Brain Injury

*Preparation before data generation*: Most existing studies have only simulated the strain field of local tissues caused by individual probes of BCI, neglecting potential global injury and the interactions between adjacent probes. In contrast, predicting BCI caused whole brain strain offers a more comprehensive assessment of the injury and preserves the operating space for expanding to more challenging conditions, such as patient implanted interacting multiple BCI (multiple probe arrays). Nevertheless, BCI caused whole brain injury simulation is more complex, time‐consuming, difficult to subject‐specific analysis and has been rarely researched.^[^ ^44^, ^52^ ^]^ Thus, we construct the simulator that simulates the injury of the whole brain implanted probe array, to generate data for training efficient, patient‐specific models for whole brain injury prediction.

To the best of our knowledge, this study represents one of the first attempts to model whole‐brain injury induced by BCI, with the aim of providing a more comprehensive framework for injury quantification while ensuring flexibility and scalability for future research. Although direct validation using an identical model remains unavailable, we have made substantial efforts to align our model configuration with established studies—drawing from existing literature on brain CAD models, BCI types, geometric and material properties, simulation techniques, and data sampling strategies. By rigorously incorporating detailed material characteristics, geometric representations, and mechanical behaviors, our simulations strive to more accurately capture the complex interactions between BCI and brain tissue, thereby enhancing the realism and reliability of the computational predictions. Specifically, the brain CAD model used in this study was adopted from the publicly available resource: https://github.com/Mobina‐Zibandepour/Brain‐3D‐Cad‐Model. Considering several models of probe of BCI, the adaptive and multi‐functional hydrogel probe^[^ ^63^ ^]^ is finally selected due to its good biological compliance and powerful functions. And it is extended it to 5 × 5 probe array which enables to measure and stimulate a group of neuron, to consider the possible stack effect of several adjacent probes on the whole brain.

In order to simulate the damage caused by the relative movement between the implanted and fixed probe and the whole brain while decoding the signals of the brain motor area, we construct the CAD model of the brain with the implanted probe based on Boolean operations.^[^ ^66^ ^]^ In this study, we implanted the hydrogel probe array into the frontal lobe of the brain from above to simulate the injury caused by the brain computer interface in monitoring and regulating electrical signals in the brain motor areas for patients with movement disorders. It is worth noting that this methodology is not only applicable to the above situation, but can also meet diverse medical needs as the material properties, geometrical information, and implantation position of implants can be flexibly changed.

*Data generation*: Through adaptive finite element mesh generation, we get the model consisting of over 300 000 elements that can be used for finite element calculations. Then, the nonlinear finite element simulator will sample and perform high‐fidelity calculations on multiple sets of inputs according to the requirements of data generation 1 and data generation 2, respectively. In each generation, we generate 900 sets of samples, of which 80% were used for training and 20% for validation.

In data generation 1, we will provide two predictors with datasets simulating brain injury during simulated respiration and vascular pulsation, respectively, which are representative physiological activities. This is achieved by setting different brain micromotion separately. To ensure that the predictors are general and effective for human, we set the nearly incompressible whole brain elastic modulus*E_brain_* = 9*kPa*, Poisson's ratioµ_*brain*_ = 0.45, coefficient of variation of the elastic modulus*v_brain_* = 0.2based on statistics.^[^ ^53^, ^54^, ^55^ ^]^ Each probe in probe array is set elastic modulus*E_probe_* = 16.5 kPa, Poisson's ratioµ_*probe*_ = 0.49, coefficient of variation of the elastic modulus*v_probe_* = 0.2_._^[^ ^67^, ^68^ ^]^ In order to simulate relative motion caused injuries, we first set the boundary conditions of the whole brain by fixing the bottom grid of the brainstem.^[^ ^62^, ^79^ ^]^ And considering the combined effects of long‐term aperiodic drift and physiological activity, the initial displacement of probe array was set respectively^[^ ^14^, ^22^, ^24^, ^27^ ^]^ to describe the relative motion between skull and whole brain during main physiological processes (i.e., respiration and vascular pulsation). During this processes, large deformation caused by mutual squeeze between brain tissue and hydrogel probe array is the source of nonlinearity. The above setting ensures a balance^[^ ^64^, ^69^ ^]^ between accuracy and efficiency in data generation 1. Data generation 2 focuses on patient‐specific injury descriptions in various actions under several noise levels. In order to simulate the brain injury caused by brain micromotion due to various actions in daily life, we modeled the displacement of the probe array as a Gaussian distribution and set the mean *u* = 0 and standard deviation σ = 100µ*m* ^[^ ^14^, ^22^, ^63^, ^65^ ^]^ in three mutually perpendicular directions as possible brain micromotion and simulate the whole brain von Mises strain fields. The properties of patient brain and probe array are set by the “optimal choice” which records the properties of the patient brain and the BCI with minimize injury to the patient.

Subsequently, the simulator will automatically calculate the output (brain injury) based on each set of input to generate high‐fidelity datasets for the training of general injury prediction module and real‐time monitoring module. Generally, the finite element calculations in data generation 1 and data generation 2 aim to solve the following problems: a structure that occupies the undeformed domain *x* ∈ Ω⊂*R* ^3^, which is subject to boundary conditions over the boundary Γ, as well as a volumetric body force, *f*(*x*): Ω → *R* ^3^. Under these conditions the structure undergoes a deformation *u*(*x*): Ω → *R* ^3^, and depending the discretisation strategy, the deformation is represented by the finite dimensional vector *u* ∈ *R^N^*, satisfying the general set of nonlinear equilibrium equations

(5) F(θ,u)=0

where θ ∈ *X*⊂*R^s^* represents the parameterisation of the problem. The parameterisation, may include spatially varying loading conditions, material parameters or perhaps uncertain boundary conditions.

The problem we solve includes large deformation which requires nonlinear theory.^[^ ^70^ ^]^ When calculating the output for each set of inputs, we consider dividing the complete load (displacement) into several parts, and loading them sequentially, which is called incremental finite element method^[^ ^70^ ^]^:

(6) $$K_{T}dU=dF$$

where *K_T_* denotes the tangent stiffness matrix, *d* **U** is the node displacement increment vector, and *d* **F** is the node force increment vector. We use the Newton‐Raphson method.^[^ ^71^, ^72^ ^]^ which is the most frequently scheme as a numerical technique for solving the equilibrium equation of the nonlinear problem, to solve a series of linear equations. More details on solving the nonlinear problem, including description of boundary value problem, development of weak form, discretization, linearization, and Newton iteration method will be presented in nonlinear theory.^[^ ^70^ ^]^

After completing Data Generation 2, we introduced additional noise into the original dataset to more accurately reflect real‐world conditions and assess the long‐term robustness of the proposed system.^[^ ^86^, ^87^, ^88^ ^]^ Specifically, we incorporated various noise levels to emulate potential data degradation and transmission errors that may arise in practical applications. We then evaluated the predictive performance of the proposed model across three datasets: the original noise‐free data, the medium‐noise data, and the high‐noise data. This comparative analysis enables a systematic assessment of the model's stability and accuracy under varying data quality conditions, offering valuable insights into its robustness and reliability for deployment in real‐world scenarios.

### Feature‐Based Predictor/Monitor

We present the feature‐based emulator (predictor/monitor), constructed by Gaussian Process emulators and model reduction technique. As a data‐driven scheme, it avoids time‐consuming processes such as assembling stiffness matrices and solving a system of equations in data generation 1 and data generation 2. Additionally, the proposed framework is based on Bayesian theory.^[^ ^47^, ^48^ ^]^ providing more robust and reliable modeling results along with the kernel functions with naturally evaluation and processing of different noise. This allows for accurate and quite efficient predictions of brain injuries.

*Feature Capturing scheme for High‐Dimensional Brain Injury*: The proposed framework utilizes model reduction technique, specifically principal component analysis.^[^ ^73^, ^74^ ^]^ to construct a feature space that retains the features of full‐field output (i.e., brain injury) obtained from the nonlinear finite element simulator in each computation with minimal loss of information.^[^ ^75^ ^]^ Principal component analysis has been widely adopted in various fields due to its ability to efficiently approximate dimension reduction for large‐scale datasets while minimizing information loss.^[^ ^76^, ^77^, ^78^ ^]^

Consider that we have sampled the required *M* sets of inputs ε and calculated the sets of outputs ζ corresponding to the inputs from the nonlinear finite element simulator:

(7) $$\varepsilon =\left[\varepsilon^{\left(1\right)},\varepsilon^{\left(2\right)},\text{…},\varepsilon^{\left(M\right)}\right]\in R^{I\times M}$$

(8) $$\zeta =\left[\xi^{\left(1\right)},\xi^{\left(2\right)},\text{…},\xi^{\left(M\right)}\right]\in R^{J\times M}$$

where ε^(*K*)^ is the output corresponding to ξ^(*K*)^(*K* = 1, #x02026;, *M*), *I* and *J* are dimensions of a set of the input and output respectively. Here, we will reduce the dimension of the outputs using principal component analysis. We first centre the output data such that:

(9) $$\hat{\zeta }=\left[\xi^{\left(1\right)}-\mu_{\xi },..,\xi^{\left(M\right)}-\mu_{\xi }\right]$$

where $\mu_{\xi }=\sum_{j=1}^{M}\xi^{\left(j\right)}$, then we compute the single value decomposition of $\hat{\zeta }$, such that:

(10) $$\hat{\zeta }=\Phi DV$$

*D* being a diagonal matrix of (decreasing) singular values of $\hat{\zeta }$ and the columns of Φ and the columns of *V* are called the left and right‐singular vectors of $\hat{\zeta }$, respectively. The singular values present the contribution of each eigenvector to the variance observed in $\hat{\zeta }$ in describing the whole dataset. Thus, a truncated / reduced representation of $\hat{\zeta }$ can be defined as

(11) $$\tilde{\Phi }=\left[\Phi_{1},\Phi_{2},\text{…},\Phi_{m}\right]\in R^{J\times m}$$

where *m* designates the dimension of the reduction space. In most samples, we set the truncation error to a certain value (less than 10^−10^) to preserve as much information in the raw dataset as possible in the feature space, and *m*(*m* ≪ *M*) is determined. We will apply the above order reduction model in the proposed predictor to hunt for the reduction space of the dataset we are interested in in this paper:

(12) $$\xi \left(\omega \right)=\mu_{\zeta }+\sum_{k=\alpha_{i}}^{m}\alpha_{i}\left(\omega \right)\Phi_{j}$$

Note in particular that the eigen‐vectors here are ortho‐normal, i.e., Φ_*i*_ · Φ_*j*_ = 0 if *i* ≠ *j*. For a given sample ω(*j*) and the principal component Φ_*k*_:

(13) $$\left(\xi \left(\omega^{\left(j\right)}\right)-\mu_{\zeta }\right)\cdot \Phi_{k}=\left(\sum_{k=i}^{m}\beta_{i}\left(\omega^{\left(j\right)}\right)\Phi_{j}\right)\cdot \Phi_{k}=\beta_{k}\left(\omega^{\left(j\right)}\right)$$

*Feature‐based Predictor/Monitor*: We build the feature‐based predictor/monitor based on Gaussian process emulator. It is assisted by a feature capturing scheme mentioned above to capture the mapping of the input $\varepsilon \in R^{I}$ and the features $\beta \in R^{r}$ of output $\xi \in R^{J}$, to enhance the performance of Gaussian process emulator for high‐dimensional predictions, and the workflow in Figure 3 of the predictor/monitor can be briefly described as follows:

(14) $$\varepsilon \in R^{I}\underset{Emulate}{\Rightarrow }\beta \in R^{r}\underset{\mathrm{Pr}oject}{\Rightarrow }\xi \in R^{J}$$

For the “Emulate” in the workflow, we build *r* independent Gaussian Processes from the input space 𝜺 with each principal component coefficient β_*k*_ (scalar):

(15) $$\beta_{k}∼GP\left(\mu_{k}\left(\varepsilon \right),C_{k}\left(\varepsilon ,\varepsilon^{\prime }\right)\right)\begin{array}{ccc} & for & k=1,\text{…},r \end{array}$$

where µ_*k*_ represents the mean function. *C_k_* is the covariance function which is diverse, depending on the regression requirements specific to the problem:

(16) $$C_{k}=\left[\begin{array}{ccc} c_{k}\left(x_{1},x_{1}\right) & \text{…} & c_{k}\left(x_{1},x_{d}\right)\\ \vdots & \ddots & \vdots \\ c_{k}\left(x_{d},x_{1}\right) & \text{…} & c_{k}\left(x_{d},x_{d}\right) \end{array}\right]\in \;R^{d\times d}$$

where *d* is the dimension of input space 𝜺. In this study, the Gaussian kernel, known as the radial basic function (RBF) kernel, is utilized to match the full‐field prediction of brain injury:

(17) $$c_{k}\left(x_{i},x_{j}\right)=\sigma_{k}^{2}exp\left(-\frac{|x_{i}-x_{j}{|}^{2}}{2l^{2}}\right)$$

The kernel function with the hyperparameters $\sigma_{k}$ and *l* in Gaussian process which describes the correlation between different data points within the input space 𝜺 should be appropriately determined. Through comprehensive experiments and considerations, we realize the selection and optimization of hypermeters by using the scheme of maximizing marginal likelihood strategy.

We first split the data (ε and β) into training data (ε_1_ and β_1_) used to train the proposed predictor/monitor whose size is *n*_1_ and the testing data (ε_2_ and β_2_ with a size of *n*_2_ for testing, and the total number of samples *M* = *n*_1_ + *n*_2_. In this scheme, we hope maximize the likelihood of the presence of the output β_1_ in training data after observing the input in training data ε_1_ through continuously searching for the optimal hyperparameters. The conditional probability density of output β_1_, called the marginal likelihood, can be written as:

(18) $$p\left(\beta_{1}|\varepsilon_{1},\theta \right)=\frac{1}{\sqrt{{\left(2\pi \right)}^{d}|C_{k}\left(\theta \right)}}\exp\left(-\frac{1}{2}{\beta_{1}}^{T}C_{k}{\left(\theta \right)}^{-1}\beta_{1}\right)$$

where $\theta ={\left[\begin{array}{cc} \sigma_{k} & l \end{array}\right]}^{T}$ is the hyperparameter vector. Usually, its log form is employed to simplify the computation of hyperparameter optimization:

(19) $$\log p\left(\beta_{1}|\varepsilon_{1},\theta \right)=-\frac{1}{2}{\beta_{1}}^{T}C_{k}{\left(\theta \right)}^{-1}\beta_{1}-\frac{1}{2}\log\left|C_{k}\left(\theta \right)\right|-\frac{d}{2}\log\left(2\pi \right)$$

In this form, the term $-\frac{1}{2}{\beta_{1}}^{T}C_{k}{\left(\theta \right)}^{-1}\beta_{1}$ in right‐hand side presents the empirical risk which measures the difference between predictions and observations; the term $\frac{1}{2}\log\left|C_{k}\left(\theta \right)\right|$ serves as the regularization term, or penalty for the complexity to prevent overfitting. We will minimize its negative log form *L*(θ) = −log *p*(β_1_|ε_1_,θ), which transforms the search for hyperparameters into an unconstrained optimization process:

(20) $$\hat{\theta }=\underset{\theta }{\arg\min}L\left(\theta \right)$$

The conjugate gradient descent method, which provides a both computational and memory efficient, fast convergence, and highly adaptable approach for optimizing hyperparameters in Gaussian process regression. The initial search direction can be set as:

(21) $$g_{0}=-\nabla L\left(\theta_{0}\right)$$

where θ_0_ is the initial vector of hyperparameters. In the *n*‐th iteration, hyperparameters will be further searched:

(22) $$\theta_{n+1}=\theta_{n}+a_{n}g_{n}$$

where *a_n_* presents the step length through line search, which minimize the objective function *a_n_*:

(23) $$\alpha_{n}=\underset{\alpha }{\arg\min}L\left(\theta_{n}+\alpha g_{n}\right)$$

Then the search direction in next iteration will be updated by the Polak‐Ribi`ere formula:

(24) $$g^{n+1}=-\nabla L^{n+1}+\frac{{\left(\nabla L^{n+1}\right)}^{T}\left(\nabla L^{n+1}-\nabla L^{n}\right)}{{\left(\nabla L^{n}\right)}^{T}\nabla L^{n}}g^{n}$$

In this process, *L* is regarded as a function of $\theta ={\left[\begin{array}{cc} \sigma_{k} & l \end{array}\right]}^{T}$, so its gradient ∇*L* can be written as:

(25) $$\nabla L=\frac{\partial L}{\partial \sigma_{k}}\cdot \vec{i}+\frac{\partial L}{\partial l}\cdot \vec{j}$$

where

(26) $$\left\{\begin{array}{c} \frac{\partial L}{\partial \sigma_{k}}=\frac{1}{2}{\beta_{1}}^{T}{C_{k}}^{-1}\frac{\partial C_{k}}{\partial \sigma_{k}}{C_{k}}^{-1}\beta_{1}+\frac{1}{2}tr\left({C_{k}}^{-1}\frac{\partial C_{k}}{\partial \sigma_{k}}\right)\\ \frac{\partial L}{\partial l}=\frac{1}{2}{\beta_{1}}^{T}{C_{k}}^{-1}\frac{\partial C_{k}}{\partial l}{C_{k}}^{-1}\beta_{1}+\frac{1}{2}tr\left({C_{k}}^{-1}\frac{\partial C_{k}}{\partial l}\right) \end{array}\right.$$

The optimal hyperparameters are searched by the iterations mentioned above until the convergence condition is satisfied. The criteria usually is set as the norm of the gradient:

(27) $$∥\nabla L(\theta_{k+1})∥<\varepsilon$$

It is worth noting that we have established Gaussian processes regression with hyperparameter optimization for each principal component, which presents flexible function space descriptions for principal components with both efficiency and flexibility, making the framework suitable for handling high‐dimensional problems.

Here we initialize the mean µ(ε) = 0 and covariance using exponentiated quadratic kernel function. Then, we will perform posterior predictions based on Bayesian theory. To predict the expected value and probability of the output variable β_2_ for a new set of test input ε_2_, we condition the observed data on our created posterior distribution *p*(β_2_|β_1_,ε_1_,ε_2_) by Bayesian theory. The training output β_1_ and the testing output β_2_ are jointly Gaussian distributed and since they both come from the same distribution, we have:

(28) $$\left[\begin{array}{c} \beta_{1}\\ \beta_{2} \end{array}\right]∼N\left(\left[\begin{array}{c} \mu_{1}\\ \mu_{2} \end{array}\right],\left[\begin{array}{cc} \sum_{11} & \sum_{12}\\ \sum_{21} & \sum_{22} \end{array}\right]\right)$$

where

(29)

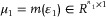



(30)

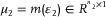

(31) $$\sum_{11}=C\left(\varepsilon_{1},\varepsilon_{1}\right)\in R^{n_{1}\times n_{1}}$$

(32) $$\sum_{22}=C\left(\varepsilon_{2},\varepsilon_{2}\right)\in R^{n_{2}\times n_{2}}$$

(33) $$\sum_{12}=C\left(\varepsilon_{1},\varepsilon_{2}\right)\in R^{n_{1}\times n_{2}}$$

Subsequently, we can then get the conditional distribution for the reduced basis coefficient output of prediction data:

(34) $$p\left(\beta_{2}|\beta_{1},\varepsilon_{1},\varepsilon_{2}\right)=N\left(\beta_{2}|\mu_{2|1},\sum_{2|1}\right)$$

where the mean µ_2|1_ = ∑_21_∑_11_^−1^β_1_ and the variance ∑_2|1_ = ∑_22_ − ∑_21_∑_11_^−1^∑_12_.

For long‐term robustness of information systems, the proposed framework further considers the inherent errors in data transmission. These drifts are typically modeled as Gaussian noise. We consider adding Gaussian noise *v* ∼ *N*(0, σ) to the raw dataset here. Then, we can write down the jointly Gaussian distributed of the training output β_1*_ and the test output β_2*_:

(35) $$\begin{array}{c} \left[\begin{array}{c} \beta_{1*}\\ \beta_{2*} \end{array}\right]=\left[\begin{array}{c} \beta_{1}\\ \beta_{2} \end{array}\right]+\left[\begin{array}{c} v_{1}\\ v_{2} \end{array}\right]∼N\left(\left[\begin{array}{c} \mu_{1}\\ \mu_{2} \end{array}\right],\left[\begin{array}{cc} \Sigma_{11}+\sigma^{2}I & \Sigma_{12}\\ \Sigma_{12}^{T} & \Sigma_{22}+\sigma^{2}I \end{array}\right]\right) \end{array}$$

Therefore, we can also compute the conditional distribution β_2*_ ∼ *N*(µ_*_,Σ_*_) based on Bayesian theory under noise:

(36) $$\mu_{*}=\sum_{21}{\left(\sum_{11}+\sigma^{2}I\right)}^{-1}\beta_{1*}$$

(37) $$\sum_{*}=\sum_{22}+\sigma^{2}I-\sum_{21}{\left(\sum_{11}+\sigma^{2}I\right)}^{-1}\sum_{12}$$

Finally, we project back to obtain the expectations and variances of the raw high‐dimensional output (the strain field of the whole brain) via

(38) $$E\left[\xi \right]=\mu_{\zeta }+\tilde{\Phi }E\left[\beta \right]$$

## Conflict of Interest

The authors declare no conflict of interest.

## Supporting information



Supporting Information

## Data Availability

The brain models that support the findings of this study are openly available at https://github.com/Mobina‐Zibandepour/Brain‐3D‐Cad‐Model, and no restrictions apply to access them. Any additional materials used in this study are available from the corresponding author upon reasonable request.
