# Supplementary material for: Reliable AI Platform for Monitoring BCI Caused Brain Injury and Providing Real‐Time Protection
Source: Adv Sci (Weinh). 2025 Dec 24;13(7):e06747. doi: 10.1002/advs.202506747 (PMC12866877; doi:10.1002/advs.202506747)
Supplement: Supplementary file 1 — Supporting Information [file ADVS-13-e06747-s001.docx]

Supplementary Information for BrainGuard

**Chufan He ^a, #^, Yanjun Ding ^b, #^, Timon Rabczuk ^c^, Chensen Ding ^a,^ ***

*^a^ School of Mechanics and Engineering Science, Peking University, Beijing, 100871, China.*

*^b^ Xiangya School of Basic Medical Sciences, Central South University, Changsha, 410013, China.*

*^c^ Chair of Computational Mechanics, Bauhaus University Weimar, Weimar, 99423, Germany.*

**Contents**

1 **Supplementary results of brain injury during respiration**
2 **Supplementary results of brain injury during vascular pulsation**
3 **Supplementary real-time monitoring results of brain injury in various noise levels**
4 **FEM simulator validation**
5 **Assessment and selection algorithm**

**1 Supplementary results of brain injury during respiration**

To provide a more comprehensive display of the predicted results of brain injury during **respiration**, we draw several cross-sectional views in two mutually perpendicular directions, respectively, to demonstrate the excellent prediction performance within the brain. The error between prediction and reference is less than 0.0005 independent on the strain levels in the entire brain, which is crucial for patient injury assessment. For a clearer presentation, we divide the Fig.1 into two parts.

**(Supplementary Fig.1 Part1)**

**(Supplementary Fig.1 Part2)**

**Supplementary Fig.1 Prediction result of brain injury during respiration within the brain: several cross-section views in two mutually perpendicular directions.** Comparisons of prediction and reference for each view, as well as relative errors. X and Y represents the clip position of the views, and the red arrow represents the viewing angle.

**2 Supplementary results of brain injury during vascular pulsation**

We additionally provide several cross-sectional views of the brain to provide a more comprehensive display of the prediction of brain injury during **vascular pulsation**. The prediction and reference were consistent, and the error is less than 3.3×10^-4^.

**(Supplementary Fig.2 Part1)**

**(Supplementary Fig.2 Part2)**

**Supplementary Fig.2 Prediction result of brain injury during vascular pulsation within the brain: several cross-section views.** Comparisons of prediction and reference for each image, as well as relative errors. X and Y represents the clip position of the views, and the red arrow represents the viewing angle.

**3 Supplementary real-time monitoring results of brain injury**

We supplement the excellent results of **real-time monitoring under various noise levels (without noise, 10dB SNR and 5dB SNR)** in the main text, including predictions, references and errors of brain injury within the brain, presented in cross-sectional views. For a better presentation, we will divide the Fig.3 into two parts.

**(Supplementary Fig.3 Part1)**

**(Supplementary Fig.3 Part2)**

**Supplementary Fig.3 Real-time monitoring result of brain injury in daily activity within the brain: multiple cross-sectional images (Without noise).** Comparisons of prediction and reference for each image, as well as relative errors. X represents the position of the profile, and the red arrow represents the viewing angle.

It can be observed that the whole brain prediction of the proposed framework is very consistent with the reference, which demonstrates that it can make accurate and efficient predictions without noise.

**(Supplementary Fig.4 Part1)**

**(Supplementary Fig.4 Part2)**

**Supplementary Fig.4 Real-time monitoring result of brain injury in daily activity within the brain: multiple cross-sectional images (With 10dB noise).** Comparisons of prediction and reference for each image, as well as relative errors. X represents the position of the profile, and the red arrow represents the viewing angle.

Subsequently, we display accurate predictions of the proposed monitor at various noise levels (10dB in Fig. 4, 5dB in Fig. 5) at each view. It demonstrates that the proposed framework can effectively evaluate different levels of noise and achieve efficient prediction of whole brain injury. BrainGuard has great potential to further enhance the long-term robustness of the BCI system integrated with it.

**(Supplementary Fig.5 Part1)**

**(Supplementary Fig.5 Part2)**

**Supplementary Fig.5 Real-time monitoring result of brain injury in daily activity within the brain: multiple cross-sectional images (With 5dB noise).** Comparisons of prediction and reference for each image, as well as relative errors. X represents the position of the profile, and the red arrow represents the viewing angle.

Figs. 6 and 7 extend the analysis to biased Gaussian noise (mean = 0.001 and 0.005, SNR = 5 dB), providing a more stringent test than the zero-mean cases. Across multiple sagittal and coronal slices, BrainGuard predictions remain closely aligned with the reference strain fields, with most errors confined to low levels. Although stronger deviations appear under the 0.005 bias compared to the 0.001 case, the dominant strain patterns are still well preserved. These findings demonstrate that the framework can effectively manage the combined effects of systematic bias and strong disturbance, maintaining robust performance and ensuring accurate strain field estimation even under severe biased noise.

**(Supplementary Fig.6 Part1)**

**(Supplementary Fig.6 Part2)**

**Supplementary Fig.6 Real-time monitoring result of brain injury in daily activity within the brain: multiple cross-sectional images (Gaussian noise mean=0.001, SNR=5dB).** Comparisons of prediction and reference for each image, as well as relative errors. X represents the position of the profile, and the red arrow represents the viewing angle.

**(Supplementary Fig.7 Part1)**

**(Supplementary Fig.7 Part2)**

**Supplementary Fig.7 Real-time monitoring result of brain injury in daily activity within the brain: multiple cross-sectional images (Gaussian noise mean=0.005, SNR=5dB).** Comparisons of prediction and reference for each image, as well as relative errors. X represents the position of the profile, and the red arrow represents the viewing angle.

**4 FEM simulator validation**

Supplementary Fig. 8 shows that the BrainGuard’s simulator produces strain distributions around hydrogel, polycarbonate (PC), and stainless-steel implants in breathing settings that closely follow the patterns seen in both FEM results and experimental biocompatibility data in reference. The variations likely reflect differences in geometric models, loads, computation methods, and numerical accuracy in machine.

Hydrogel probes generate the lowest strain near the probe–tissue interface, which matches the reduced expression of IgG, GFAP, CD68, and Iba1 reported in the biological experiments. By contrast, PC and stainless-steel probes create very similar patterns, with several regions of higher strain around the implantation site. This is explained by their weaker coupling with brain tissue: because of lower friction, these probes are more likely to slip or collide with the surrounding tissue, producing stronger micromotion-related stress. BrainGuard simulator reveals a clearer trend: the steel probes show a slightly darker overall color, indicating higher strain, which aligns with the stronger immune and glial responses seen experimentally (IgG, GFAP, CD68, Iba1).

These findings show that the BrainGuard’s simulator effectively captures tissue-probe interactions material-dependent differences consistent with reference data. With this validation, the simulator can be used to generate high-fidelity datasets that support data-driven modeling of brain–implant interactions.

**Supplementary Fig.8 Comparison of BrainGuard’s simulator and Park’s results [11].**

To verify the applicability of FEM-generated data with the experimental results, we first reconstructed the statistical perspective of the variation curve of spiking electrodes (unit yield) over time by using the data of unit yield in BrainGate from all patient sampling points at all measurement times, as well as the long-term trend curve (light green box in the figure, titled 'spiking electrodes data in BrainGate'). Then, from each of the 900 FEM simulations under different micromotions, we compute full-field strain and focus on the area near the implantation site, where the damage was most severe (Fig. 9). A logistic damage function is modeled and applied to convert local strain into neuronal loss probability [1][2]:

| $P_{1oss}(\varepsilon)=\frac{1}{1+exp\left[-\alpha\left(\varepsilon-\varepsilon_{crit}\right)\right]}$ | (1) |
| --- | --- |

with *α*=30 for effectively description of the rapid evolution of injury from scratch [3][4], and *ε_crit_*=0.05 as a conservative threshold to ensure that subtle but cumulative neuronal damage is not underestimated [5][6]. And the unit yield (fraction of surviving neurons) [4][7][8] was obtained as:

| $Y=\frac{1}{V}\int_{V} (1-P_{1oss}(\varepsilon))dV$ | (2) |
| --- | --- |

where *V* is a volume very close to the implantation area, as shown in Fig. 9. Then, we aligned BrainGuard's FEM-based spike electrode data (88% → 60%) with the BrainGate benchmark by scaling and setting the initial values to the same starting point as the clinical data (41%). This adjustment accounts for the inherent biocompatibility advantages of hydrogel electrodes, which generate significantly lower strain and thus higher absolute yield compared to rigid Utah arrays [9][10][11]. By focusing on relative decline rather than absolute values, we directly assess whether FEM predictions reproduce the degradation observed in vivo.

In Fig. 10, the similarity in the overall declining trend and range validates the simulator, while the smoother trajectory across 900 simulations reflects dense and comprehensive electrode–tissue interactions, in contrast to the fluctuations shown in BrainGate recordings. Moreover, the normalized FEM-predicted decline in unit yield (41% → 28%) spans a slightly broader interval than the BrainGate benchmark (41% → 33%), which shows that the FEM-generated dataset captures not only the gradual yield loss from micromotion-driven cumulative damage but also the sharper drops occasionally observed in chronic implants. Importantly, the broader decline range does not contradict the intrinsic biocompatibility of hydrogel electrodes but instead reflects a conservative coverage of potential damage. In conclusion, these results confirm the reliability of the FEM simulator, as it reproduces the observed decline trend and provides a wider coverage that accounts for both gradual and abrupt yield loss, supporting its use as a trustworthy dataset for data-driven modeling when direct clinical ground truth is unavailable.

**Supplementary Fig.9 Main injury field of whole brain.**

**Supplementary Fig.10 Comparison of FEM-predicted and BrainGate-measured unit yield.** Unit yield derived from 900 FEM simulations (BrainGuard, red) is compared with averaged clinical data from 20 Utah arrays in the BrainGate trial (green). The normalized FEM-based results match the unit yield range reported in BrainGate (41%→33.6%). The comparison highlights that the predicted decline range (red vertical bar) overlaps with the clinical range observed in BrainGate (green vertical bar), indicating that FEM-generated dataset effectively reflects the inherent degradation trends in long-term neural recordings.

In Fig. 11, the relationship between dSNR and the number of spiking electrodes was derived from BrainGate clinical data. Two regression models were employed: 1) a linear fit after excluding abnormal data (brown points, mentioned in BrainGate) [12]:

| $dSNR=k\cdot N_{total}+b$, $N_{total}=Y\cdot N_{BrainGate}$ | (3) |
| --- | --- |

with minimize the linear regression loss:

| $\text{Loss}(k,b)=\frac{1}{n}\sum_{i=1}^{n} (dSNR_{i}-(k\cdot N_{total,i}+b))^{2}$ | (4) |
| --- | --- |

where *N_BrainGate_*=96 reported in reference. And a logarithmic fit that captures the nonlinear scaling between neuronal yield and SNR [13][14]:

| $dSNR=k\cdot\log(1+Y\cdot N_{total})+b$ | (5) |
| --- | --- |

with minimize the log regression loss:

| $\text{Loss}(k,b)=\frac{1}{n}\sum_{i=1}^{n} (dSNR_{i}-(k\cdot\log(1+Y_{i}\cdot N_{total})+b))^{2}$ | (6) |
| --- | --- |

By applying these models, FEM-predicted unit yields were mapped into the dSNR and compared with BrainGate. The values (red: linear; blue: logarithmic) cluster in the upper-right region of the scatter space, corresponding to both higher spiking electrode counts and dSNR.

This shift reflects the improved compliance of hydrogel probes: low micromotion-induced strain at the tissue–electrode interface reduces microvascular injury and glial scarring, stabilizes impedance, and slows the decay of sortable units, thereby maintaining higher yield and SNR. In contrast, Utah arrays present a large mechanical mismatch with brain tissue, amplifying shear forces and leading to chronic blood–brain barrier leakage, astroglial activation, and progressive signal degradation. As a result, BrainGate data shows broader variability and lower averages. Notably, prior studies on flexible and hydrogel-based probes consistently report higher unit yield and stable SNR compared with rigid Utah arrays [9][10][11], consistent with the rightward and upward clustering of the FEM points.

**Supplementary Fig.11 Comparison of FEM-predicted and BrainGate-measured dSNR.** BrainGate data were fitted using both linear (excluding abnormal points) and logarithmic regressions for the dSNR–spiking electrode relationship. Through these models, FEM-derived values were mapped and found consistently in the upper-right region of the plot, indicating higher unit yield and dSNR compared with the Utah array data.

In summary, these findings demonstrate that the simulator in BrainGuard provides a reliable long-term description of BCI-brain interaction. By reproducing the degradation observed in BrainGate and mapping FEM-derived unit yield into the dSNR domain through both linear and logarithmic models, the simulator captures the overall declining trajectory of chronic recordings. Importantly, the clustering of FEM-derived values in the upper-right region of the dSNR–spiking electrode space reflects the material-specific advantages of hydrogel electrodes: lower micromotion-induced strain energy, reduced glial encapsulation, and more stable neuronal isolation. This mechanistic fidelity explains why the simulated decline range in Fig.10 is smoother yet broader, covering both gradual cumulative loss and sharper drops occasionally seen in clinical data. These findings demonstrate that FEM-generated datasets, by capturing the micromotion-driven mechanisms of neural degradation and aligning with long-term clinical data, provides reliable and physiologically grounded data for data-driven modeling.

**5 Assessment and selection algorithm**

The relative movement between the fixed probe and the brain will cause strains at the neural interface, which may lead to brain tissue injury and neural interface failure. We propose the assessment and selection algorithm which assesses the BCI caused patient brain injury based on prediction results of feature-based predictors. When scoring each group of BCI-patients, the algorithm will consider the patient brain injury during both respiration and vascular pulsation. The parameters in the algorithm can be set patient-specific. Then, the algorithm will record the input of the sample which has the minimum injury as the ‘optimal choice’ and transmit it to drive data generation to train the real-time monitor. The pseudocode can be found below.

**Algorithm 1** Assessment and selection algorithm

Data: Prediction results of brain injury during respiration and vascular pulsation

Result: Input with minimal injury (the ‘optimal choice’)

; //Number of measurement points

; //Number of predictions

; //the quantitative injury in a prediction

*Smax*; //the artificially set maximum strain

*Smin*; //the artificially set minimum strain

**{**

**for** // Loop each group prediction

**for** // Loop each point of prediction

**if** *Sp < Smin* // Less than the minimum value, almost no injury

*Ssum_i_ = Ssum_i_*

**else if** *Smin < Sp < Smax* // May cause injury

*Ssum_i_ = Ssum_i_ + Sp*

**else** // It will Cause serious injury, so don’t use this BCI

Set *Ssum_i_* to a large value

fprintf(‘This BCI will cause serious injury’)

break

**end**

**end**

**end**

**Find the minimum** *Ssum_min_* in {*Ssum_1_, Ssum_2_, Ssum_3_, ……, Ssum_N_*}, and **record the input** of *Ssum_min_* as the ‘optimal choice’.

**}**

The source code will be online soon.

**REFERENCE**

[1] Takhounts, E.G., Craig, M.J., Moorhouse, K., McFadden, J. and Hasija, V. Development of brain injury criteria, SAE Technical Paper, 0010(22), 2013.
[2] Sahoo, D., Deck, C. and Willinger, R., Axonal strain as brain injury predictor based on real-world head trauma simulations. In Proceedings of the IRCOBI Conference, Lyon, France IRC-15-30, 2015.
[3] Giordano C, Kleiven S. Evaluation of Axonal Strain as a Predictor for Mild Traumatic Brain Injuries Using Finite Element Modeling. Stapp Car Crash Journal, 58:29-61, 2014.
[4] Hajiaghamemar, M., Wu, T., Panzer, M.B. and Margulies, S.S. Embedded axonal fiber tracts improve finite element model predictions of traumatic brain injury. Biomechanics and modeling in mechanobiology, 19(3), 2020.
[5] Bentil, S.A. and Dupaix, R.B. Simulations of hydrogel-coated neural microelectrodes to assess biocompatibility improvement using strain as a metric for micromotion. Biomedical Physics & Engineering Express, 4(3), 2018.
[6] Bar-Kochba, E., Scimone, M.T., Estrada, J.B. and Franck, C. Strain and rate-dependent neuronal injury in a 3D in vitro compression model of traumatic brain injury. Scientific reports, 6(1), 2016.
[7] Shreiber, D.I., Bain, A.C. and Meaney, D.F. In vivo thresholds for mechanical injury to the blood-brain barrier. SAE transactions, 3792-3806, 1997.
[8] Fahlstedt, M., Meng, S. and Kleiven, S. Influence of strain post-processing on brain injury prediction. Journal of biomechanics, 110940(132), 2022.
[9] Zhou, T., Hong, G., Fu, T.M., Yang, X., Schuhmann, T.G., Viveros, R.D. and Lieber, C.M. Syringe-injectable mesh electronics integrate seamlessly with minimal chronic immune response in the brain. Proceedings of the National Academy of Sciences, 114(23), 2017.
[10] Kozai, T.D.Y., Langhals, N.B., Patel, P.R., Deng, X., Zhang, H., Smith, K.L., Lahann, J., Kotov, N.A. and Kipke, D.R. Ultrasmall implantable composite microelectrodes with bioactive surfaces for chronic neural interfaces. Nature materials, 11(12), 2012.
[11] Park, S., Yuk, H., Zhao, R., Yim, Y.S., Woldeghebriel, E.W., Kang, J., Canales, A., Fink, Y., Choi, G.B., Zhao, X. and Anikeeva, P. Adaptive and multifunctional hydrogel hybrid probes for long-term sensing and modulation of neural activity. Nature communications, 12(1), 2021.
[12] Hahn, N.V., Stein, E., BrainGate Consortium, Donoghue, J.P., Simeral, J.D., Hochberg, L.R. and Willett, F.R. Long-term performance of intracortical microelectrode arrays in 14 BrainGate clinical trial participants. *medRxiv*, pp.2025-07.
[13] Willsey, M.S., Shah, N.P., Avansino, D.T., Hahn, N.V., Jamiolkowski, R.M., Kamdar, F.B., Hochberg, L.R., Willett, F.R. and Henderson, J.M. A high-performance brain–computer interface for finger decoding and quadcopter game control in an individual with paralysis. Nature Medicine, 31(1), 2025.
[14] Barrese, J.C., Rao, N., Paroo, K., Triebwasser, C., Vargas-Irwin, C., Franquemont, L. and Donoghue, J.P. Failure mode analysis of silicon-based intracortical microelectrode arrays in non-human primates. Journal of neural engineering, 10(6), 2013.
